# *Rosa roxburghii* Tratt. enhances fracture healing through multitarget regulation of osteogenesis and angiogenesis: Integrated network pharmacology and molecular docking analysis

**DOI:** 10.1097/MD.0000000000048071

**Published:** 2026-03-13

**Authors:** Zhongxiu Wu, Pingping Luo, Hongshi Liu, Jiongnan Xu, Qifeng Ying, Jun Zhang, Zixue Xuan

**Affiliations:** a Department of Pharmacy, Zhejiang Provincial People’s Hospital Bijie Hospital, Bijie, Guizhou, China; b Plastic Surgery Center, Department of Orthopedics, Zhejiang Provincial People’s Hospital (Affiliated People’s Hospital), Hangzhou Medical College, Hangzhou, Zhejiang, China; c The Second School of Clinical Medicine, Zhejiang Chinese Medical University, Hangzhou, Zhejiang, China; d Center of Osteoporosis, Zhejiang Provincial People’s Hospital (Affiliated People’s Hospital), Hangzhou Medical College, Hangzhou, Zhejiang, China; e Department of Orthopedics, Zhejiang Provincial People’s Hospital Bijie Hospital, Bijie, Guizhou, China; f Center for Plastic & Reconstructive Surgery, Department of Orthopedics, Zhejiang Provincial People’s Hospital (Affiliated People’s Hospital), Hangzhou Medical College, Hangzhou, Zhejiang, China; g Center for Clinical Pharmacy, Cancer Center, Department of Pharmacy, Zhejiang Provincial People’s Hospital (Affiliated People’s Hospital), Hangzhou Medical College, Hangzhou, Zhejiang, China.

**Keywords:** angiogenesis, fracture healing, network pharmacology, osteogenesis, *Rosa roxburghii* Tratt

## Abstract

**Background:**

*Rosa roxburghii* Tratt. (Cili), a medicinal-edible herb predominantly distributed in Guizhou Province, exhibits diverse pharmacological properties, including anti-inflammatory, antioxidant, and cell proliferation-promoting effects. Despite its recognized pharmacological value, the therapeutic efficacy and underlying mechanisms in fracture healing remain unexplored. This study aims to systematically investigate its potential mechanisms by integrating network pharmacology and molecular docking.

**Methods:**

The components of *R. roxburghii* Tratt. were retrieved from the PubChem, CNKI, VIP, and WANFANG databases, and their corresponding targets were screened from public databases. The targets of fracture healing were obtained from the GeneCards and OMIM databases. Gene Ontology and Kyoto Encyclopedia of Genes and Genomes analyses revealed the mechanisms of *R. roxburghii* Tratt. in fracture healing. The potential targets and core components were identified by constructing a protein-protein interaction network and a series of topological networks. Finally, molecular docking was performed to validate the binding of the core targets to the components.

**Results:**

Gene Ontology analysis highlighted its regulation of vascular endothelial growth factor signaling pathway, cell migration, epidermal growth factor receptor signaling pathways, and fibroblast growth factor receptor signaling pathways. Kyoto Encyclopedia of Genes and Genomes analysis suggested that *R. roxburghii* Tratt.’s mechanism may involve key pathways, such as hypoxia-inducible factor 1, phosphoinositide 3-kinase/protein kinase B, mitogen-activated protein kinase, tumor necrosis factor, and ras-associated protein-1 signaling pathway, while topological network analysis identified 4 core targets (tumor necrosis factor, prostaglandin G/H synthase 2, epidermal growth factor receptor, and proto-oncogene tyrosine-protein kinase Src) and 21 core components (naringenin, quercetin, kaempferol, isorhamnetin, luteolin, myricetin, etc), all critically associated with osteogenesis and angiogenesis. Molecular docking confirmed strong binding between these components and targets.

**Conclusion:**

These findings propose that *R. roxburghii* Tratt. may accelerate fracture healing by multitarget, components, and pathways regulating osteogenesis and angiogenesis, providing a scientific basis for its development and utilization.

## 1. Introduction

Fracture healing is a complex biological process (BP) that involves the coordinated participation of various cellular and molecular mechanisms, including inflammation, angiogenesis, cell proliferation, and bone remodeling.^[1,2]^ Despite significant advances in orthopedic medicine, the incidence of delayed or nonunion fractures is 5% to 10%, posing a significant challenge to fracture treatment and necessitating the exploration of new strategies to enhance bone repair.^[1,3,4]^ Fracture healing is a slow and continuous process,^[5]^ during which severe cases may experience complications including nonunion, delayed union, or malunion, resulting in physical and psychological disabilities, particularly among elderly patients.^[6,7]^ These complications not only reduce the quality of life but also place a significant burden on families and the society. Traditional Chinese medicines have gained increasing attention for their potential to promote fracture healing due to their multitarget and multipathway pharmacological effects.^[8]^

*Rosa roxburghii* Tratt. (commonly known as Cili), a plant native to Guizhou Province in southwest China, has been traditionally used for its anti-inflammatory, antioxidant, and immunomodulatory properties.^[9]^ The chemical composition of *R. roxburghii* Tratt. is diverse, including flavonoids, triterpenes, organic acids, tannins, phenolic compounds, polysaccharides, carotenoids, triterpenoids, volatile compounds, amino acids, vitamin C, and essential oils.^[10,11]^ It is reported that the fresh fruit of *R. roxburghii* Tratt. is rich in total flavonoids, which possess anti-inflammatory, antioxidant, and cell proliferation-promoting pharmacological activities.^[12]^ In the fracture healing process, these compounds can accelerate fracture repair by inhibiting inflammatory responses, improving microcirculation, and enhancing the activity of osteoblasts. However, the specific mechanisms by which *R. roxburghii* Tratt. contributes to fracture healing remain poorly understood.

Recent advances in network pharmacology and molecular docking have provided powerful tools for elucidating the mechanisms of natural compounds.^[13]^ Network pharmacology integrates systems biology, bioinformatics, and pharmacology to construct topological networks, enabling the identification of key bioactive components and their associated molecular pathways.^[14]^ Molecular docking, on the other hand, allows for the prediction of binding affinities between small molecules and target proteins, offering insights into the interactions at the atomic level. Together, these approaches provide a comprehensive framework for understanding the multitarget effects of herbal medicines.

**Figure 1.**
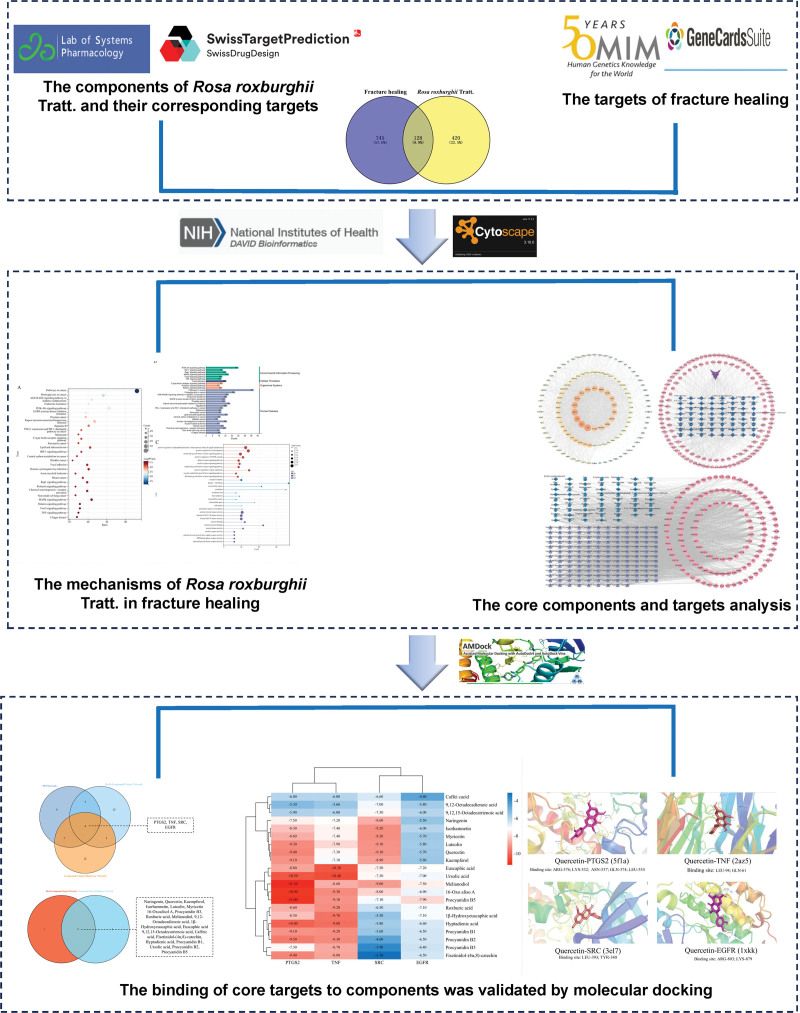
The flow of this study. EGFR = epidermal growth factor receptor, PTGS2 = prostaglandin G/H synthase 2, SRC = proto-oncogene tyrosine-protein kinase Src, TNF = tumor necrosis factor.

In this study, we aimed to investigate the potential mechanisms of *R. roxburghii* Tratt. in fracture healing using a combination of network pharmacology and molecular docking. First, we identified the active compounds of *R. roxburghii* Tratt. and their potential targets. Next, we constructed protein-protein interaction (PPI), herb-compound-target, and compound-target-pathway networks to explore the interactions between these *R. roxburghii* Tratt. and healing. Finally, molecular docking was performed to validate the binding affinities of core components to their respective targets (Fig. 1). Our findings provide new insights into the molecular mechanisms underlying the therapeutic effects of *R. roxburghii* Tratt. in fracture healing and highlight its potential as a natural remedy for bone repair.

## 2. Materials and methods

### 2.1. R. roxburghii Tratt.-related target prediction

The chemical components of *R. roxburghii* Tratt. were searched in the PubChem, CNKI, VIP, and WANFANG databases, where “Cili” and “*Rosa roxburghii* Tratt.” were selected as the subject terms, and “chemical component” was selected as the subsubject term. The simplified molecular input line entry system of the components were retrieved from the PubChem database and subsequently imported into the SwissTargetPrediction database to predict the targets of the components.

### 2.2. Fracture healing-related target screening

The targets obtained from the GeneCards database (https://www.genecards.org) and the OMIM database (https://www.omim.org/) were combined, and duplicate targets were removed.

### 2.3. Gene Ontology (GO) and Kyoto Encyclopedia of Genes and Genomes (KEGG) enrichment analyses

The targets of *R. roxburghii* Tratt. components were intersected with fracture healing-related targets using Venny 2.1 (Venny 2.1.0; Centro Nacional de Biotecnología [CNB‐CSIC], Madrid, Spain), identifying the *R. roxburghii* Tratt. targets associated with fracture healing. The common targets were then uploaded to the DAVID (Laboratory of Human Retrovirology and Immunoinformatics, Frederick National Laboratory for Cancer Research, Frederick, https://davidbioinformatics.nih.gov/) platform for GO biological function annotation and KEGG pathway enrichment analysis. The top 10 GO biological functions and the top 30 KEGG pathways were selected based on their *P* values. Visualization was performed using the Sangerbox 3.0 platform (Hangzhou Mugu Technology Co., Ltd., Hangzhou, Zhejiang, China, http://sangerbox.com/).

### 2.4. PPI network construction

We constructed a PPI network on the STRING database (http://string-db.org) by limiting the species to *Homo sapien*s and setting the confidence score to 0.4 to analyze the relationships among the common targets. The network visualization and analysis were performed using Cytoscape 3.10.0 (Cytoscape Consortium, San Diego).

### 2.5. The “herb-compound-target” and “compound-target-pathway” network construction

To investigate the therapeutic potential of *R. roxburghii* Tratt. in fracture healing, we constructed “herb-compound-target” and “compound-target-pathway” networks using Cytoscape 3.10.0 based on the identified common targets. These networks provided a comprehensive overview of how *R. roxburghii* Tratt. facilitates fracture healing. Furthermore, we employed the “Analyze Network” tool to evaluate the topological properties of the networks, which enabled us to pinpoint key active components of *R. roxburghii* Tratt. that play crucial roles in fracture healing.

### 2.6. Molecular docking

The 3D structures of prostaglandin G/H synthase 2 (PTGS2; PDB ID: 5f1a), tumor necrosis factor (TNF; PDB ID: 2az5), proto-oncogene tyrosine-protein kinase Src (SRC; PDB ID: 3el7), and epidermal growth factor receptor (EGFR; PDB ID: 1xkk) were downloaded from the PDB (https://www.rcsb.org/) database. The structures (in sdf format) of compounds were downloaded from PubChem (https://pubchem.ncbi.nlm.nih.gov/), and then, these structures were converted to 3D (mol2 format) using the ChemDraw 3D software (PerkinElmer, Inc., Waltham). Finally, the 3D structures of the targets and the compounds were imported into AMDock to perform molecular docking.

## 3. Results

### 3.1. The common targets of R. roxburghii Tratt. and fracture healing

**Figure 2.**
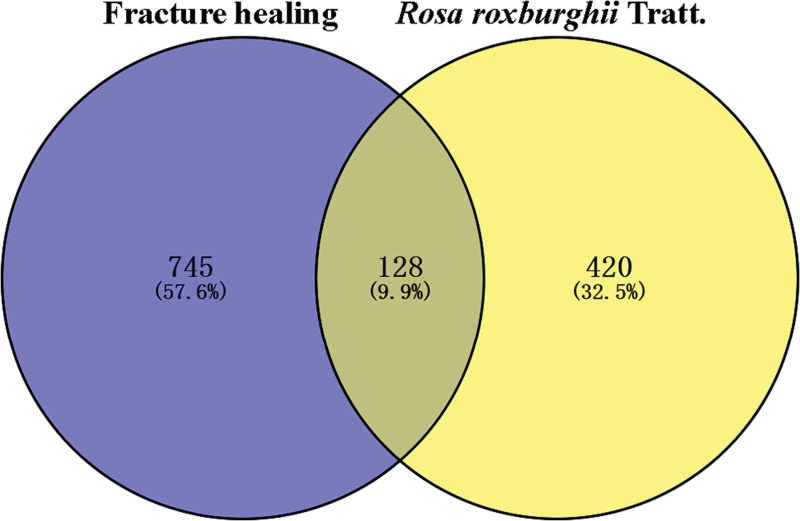
The common targets of *Rosa roxburghii* Tratt. and fracture healing.

A total of 64 components of *R. roxburghii* Tratt. were identified (Table 1), along with their corresponding 548 targets (Table S1, Supplemental Digital Content, https://links.lww.com/MD/R537). Additionally, 873 targets related to fracture healing were obtained from the GeneCards and OMIM databases. Following intersection analysis, 128 common targets were identified (Fig. 2, Table 2).

**Table 1:** Sixty-four components of *Rosa roxburghii* Tratt.

No.	Component	No.	Component	No.	Component	No.	Component
1	Vitamin C	17	Caffeic acid	33	Procyanidin A1	49	Bernardioside A
2	Tellimagrandin Ⅰ	18	Protocatechuic acid 4-O-β-glucoside	34	Procyanidin B5	50	Niga-ichigoside F1
3	Tercatain	19	Chlorogenic acid	35	Fisetinidol-(4α,8)-catechin	51	Chebuloside II
4	Quercetin	20	Veratric acid	36	p-Coumaric acid	52	Picfeltarraenin X
5	Quercitrin	21	5α-[3-(4-Hydroxyphenyl)acryloyloxy]-1,3β,4α-trihydroxycyclohexane-1β-carboxylic acid	37	Erythro-guaiacylglycerol β-sinapyl ether	53	Periplocoside
6	Isoquercitrin	22	Isoquercetin	38	Pinoresinol	54	Lactic acid
7	Rutin	23	Procyanidin B1	39	Syringic acid	55	Malic acid
8	Kaempferol	24	Procyanidin B2	40	Quinic acid	56	Citric acid
9	Isorhamnetin	25	Procyanidin B3	41	Polydatin	57	9,12,15-Octadecatrienoic acid
10	Luteolin	26	Gallotannin	42	1β-Hydroxy euscaphic acid	58	9,12-Octadecadienoic acid
11	Daidzein	27	Roxburic acid	43	Euscaphic acid	59	Xanthine
12	Procyanidin	28	Kajiichigoside F1	44	Pomolic acid	60	Rosamultin
13	Protocatechuic acid	29	Naringenin	45	Alphitolic acid	61	ROSOLIC ACID
14	Gallic acid	30	Myricetin	46	Melianodiol	62	Hyptadienic acid
15	Ellagic acid	31	Quercetin-3-O-D-xyloside	47	Ursolic acid	63	Betulinic acid
16	4-Hydroxybenzoic acid	32	Phloridzin	48	16-Oxoalisol A	64	Maslinic acid

**Table 2:** The common targets of *Rosa roxburghii* Tratt. and fracture healing.

No.	Target	No.	Target	No.	Target	No.	Target	No.	Target
1	TGFBR1	27	EGFR	53	CFTR	79	MYLK	105	RXRA
2	ALPL	28	SCN9A	54	CDK9	80	CASP3	106	KDM5C
3	IL6	29	CA2	55	IL2	81	MAPK8	107	ARG1
4	VDR	30	SRC	56	TTR	82	MTOR	108	PRKCE
5	STAT3	31	PPARG	57	MMP3	83	CDK4	109	ITGAV
6	TNF	32	SHBG	58	STAT1	84	MMP8	110	CYP3A4
7	ESR1	33	PDGFRB	59	FLT3	85	DNMT1	111	MPO
8	FGF2	34	CCND1	60	PTPN11	86	MMP12	112	HNF4A
9	VEGFA	35	ITGB1	61	IGFBP3	87	LCT	113	ACHE
10	MMP2	36	F2	62	F3	88	CREBBP	114	CSF1R
11	CASR	37	MMP14	63	IGF1R	89	F10	115	AURKA
12	MMP9	38	ELANE	64	MAPK14	90	HSD11B1	116	CTSB
13	ALB	39	PIK3R1	65	MMP7	91	PLAU	117	JAK1
14	MMP13	40	PTGS2	66	HMGCR	92	NGFR	118	MAPT
15	TLR4	41	NOS2	67	BCL2	93	PRKCD	119	G6PD
16	HRAS	42	PIK3CA	68	FGF1	94	APP	120	SLC6A4
17	MMP1	43	PIK3CG	69	JUN	95	INSR	121	LCK
18	HIF1A	44	KIT	70	PTK2	96	PPARD	122	RBP4
19	AKT1	45	MAPK1	71	COMT	97	MDM2	123	AGTR1
20	SERPINE1	46	VCP	72	CYP2C19	98	ADAM17	124	SYK
21	BRAF	47	ALK	73	P2RX7	99	CCR1	125	NOX4
22	MET	48	SLC37A4	74	KDR	100	HPSE	126	HSP90AA1
23	CYP19A1	49	ACE	75	AR	101	ERBB2	127	CYP24A1
24	FGFR1	50	MAP2K1	76	MAPK3	102	CD81	128	PIK3CD
25	TERT	51	JAK2	77	CASP8	103	NFE2L2		
26	NR3C1	52	ESR2	78	DPP4	104	PTGS1		

AKT1 = AKT serine/threonine kinase 1, EGFR = epidermal growth factor receptor, MAP2K1 = mitogen-activated protein kinase kinase 1, MAPK1 = mitogen-activated protein kinase 1, MAPK3 = mitogen-activated protein kinase 3, PIK3CA = phosphatidylinositol-4,5-bisphosphate 3-kinase catalytic subunit alpha, PIK3CD = phosphatidylinositol-4,5-bisphosphate 3-kinase catalytic subunit delta, PIK3R1 = phosphoinositide-3-kinase regulatory subunit 1, PTGS2 = prostaglandin G/H synthase 2, SRC = proto-oncogene tyrosine-protein kinase Src, TNF = tumor necrosis factor.

### 3.2. Enrichment analysis results

**Figure 3.**
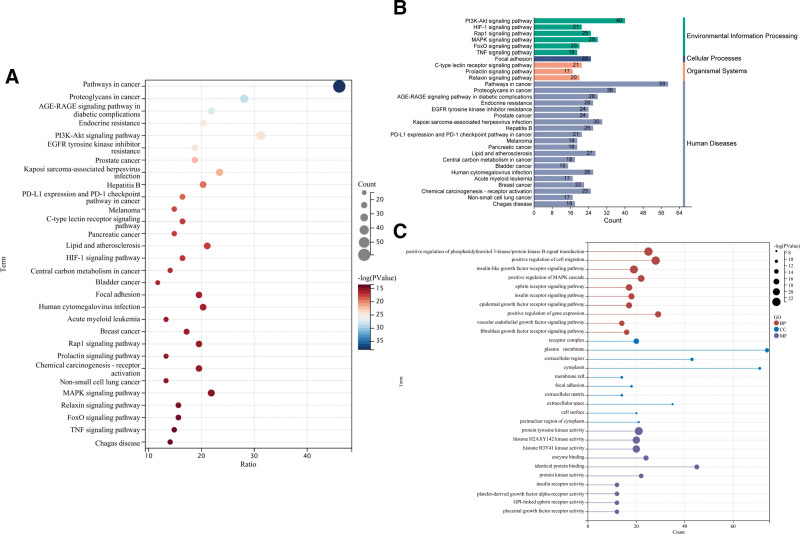
KEGG and GO enrichment analysis. (A) Bubble chart of the top 30 pathways; (B) categorized enrichment results for the top 30 pathways; and (C) lollipop plots of the top 10 terms in BP, CC, and MF from GO analysis. BP = biological process, CC = cellular component, EGFR = epidermal growth factor receptor, GO = Gene Ontology, HIF-1 = hypoxia-inducible factor 1, KEGG = Kyoto Encyclopedia of Genes and Genomes, MAPK = mitogen-activated protein kinase, MF = molecular function, PI3K-Akt = phosphoinositide 3-kinase/protein kinase B, TNF = tumor necrosis factor.

We enriched the common targets as a means of exploring the mechanisms of *R. roxburghii* Tratt. in fracture healing. KEGG pathway analysis showed that the *R. roxburghii* Tratt. involved in the expression of 153 signaling pathways (*P* value < .05; Table S2, Supplemental Digital Content, https://links.lww.com/MD/R537), and the top 30 pathways were visualized and further categorized (Fig. 3A). The environmental information processing primarily involves the following signaling pathways related to fracture healing: hypoxia-inducible factor 1 (HIF-1) signaling pathway, phosphoinositide 3-kinase/protein kinase B (PI3K-Akt) signaling pathway, mitogen-activated protein kinase (MAPK) signaling pathway, TNF signaling pathway, and ras-associated protein-1 (Rap1) signaling pathway (Fig. 3B). The GO enrichment analysis revealed that the common targets were significantly enriched in a total of 712 GO terms (*P* value < .05), including 491 BP entries (Table S3, Supplemental Digital Content, https://links.lww.com/MD/R537), 71 cellular component (CC) entries (Table S4, Supplemental Digital Content, https://links.lww.com/MD/R537), and 150 molecular function (MF) entries (Table S5, Supplemental Digital Content, https://links.lww.com/MD/R537). Among them, lollipop plots of the top 10 terms in BP, CC, and MF from GO analysis, highlighting the most significant terms in each category (Fig. 3C), and the results show that the BP analysis is mainly involved in the positive regulation of PI3K-Akt signal transduction, positive regulation of cell migration, positive regulation of MAPK cascade, vascular endothelial growth factor signaling pathway, EGFR signaling pathway, and fibroblast growth factor receptor signaling pathway. CC mainly in extracellular matrix, receptor complex, plasma membrane, MF major influence protein tyrosine kinase activity, enzyme binding, protein kinase activity.

### 3.3. PPI network of R. roxburghii Tratt. in fracture healing and key target analysis

**Figure 4.**
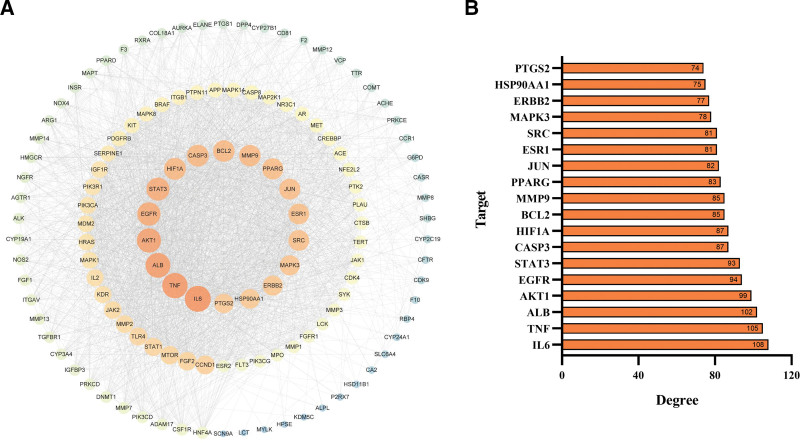
The potential key targets were identified by the PPI network. (A) PPI network and (B) 18 key targets were identified in the PPI network by selecting nodes with a degree ≥ 2-fold median. EGFR = epidermal growth factor receptor, PPI = protein-protein interaction, PTGS2 = prostaglandin G/H synthase 2, SRC = proto-oncogene tyrosine-protein kinase Src, TNF = tumor necrosis factor.

To explore the potential molecular targets of *R. roxburghii* Tratt. for fracture healing, we established a PPI network containing 125 nodes and 2624 edges based on the identified intersecting genes (Fig. 4A). The 18 key targets (degree ≥ 2-fold median) of *R. roxburghii* Tratt. that contribute to its fracture healing effect are illustrated in Figure 4B.

### 3.4. Herb-compound-target network analysis

**Figure 5.**
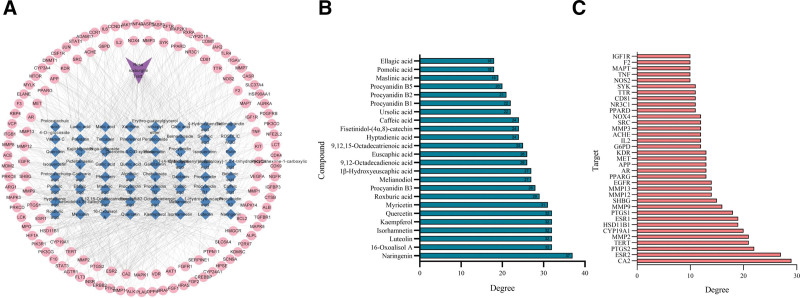
The key components and targets were identified by the herb-compound-target network. (A) The herb-compound-target network; 24 key compounds (B) and 35 key targets (C) were identified in the herb-compound-target network by selecting nodes with a degree ≥ 2-fold median. EGFR = epidermal growth factor receptor, PTGS2 = prostaglandin G/H synthase 2, SRC = proto-oncogene tyrosine-protein kinase Src, TNF = tumor necrosis factor.

The herb-compound-target network was constructed in Cytoscape 3.10.0 using *R. roxburghii* Tratt., 63 components, and 125 targets. From this, 193 nodes were obtained, and 909 interaction relationships were found (Fig. 5A). By analyzing the herb-compound-target network, the 24 compounds with the highest degree values in *R. roxburghii* Tratt. were found to be naringenin, 16-oxoalisol A, luteolin, isorhamnetin, and others, as shown in Figure 5B. In addition, we identified 35 targets with a degree value greater than or equal to twice the median degree value, further expanding our list of potential key targets (Fig. 5C).

### 3.5. Compound-target-pathway network analysis

**Figure 6.**
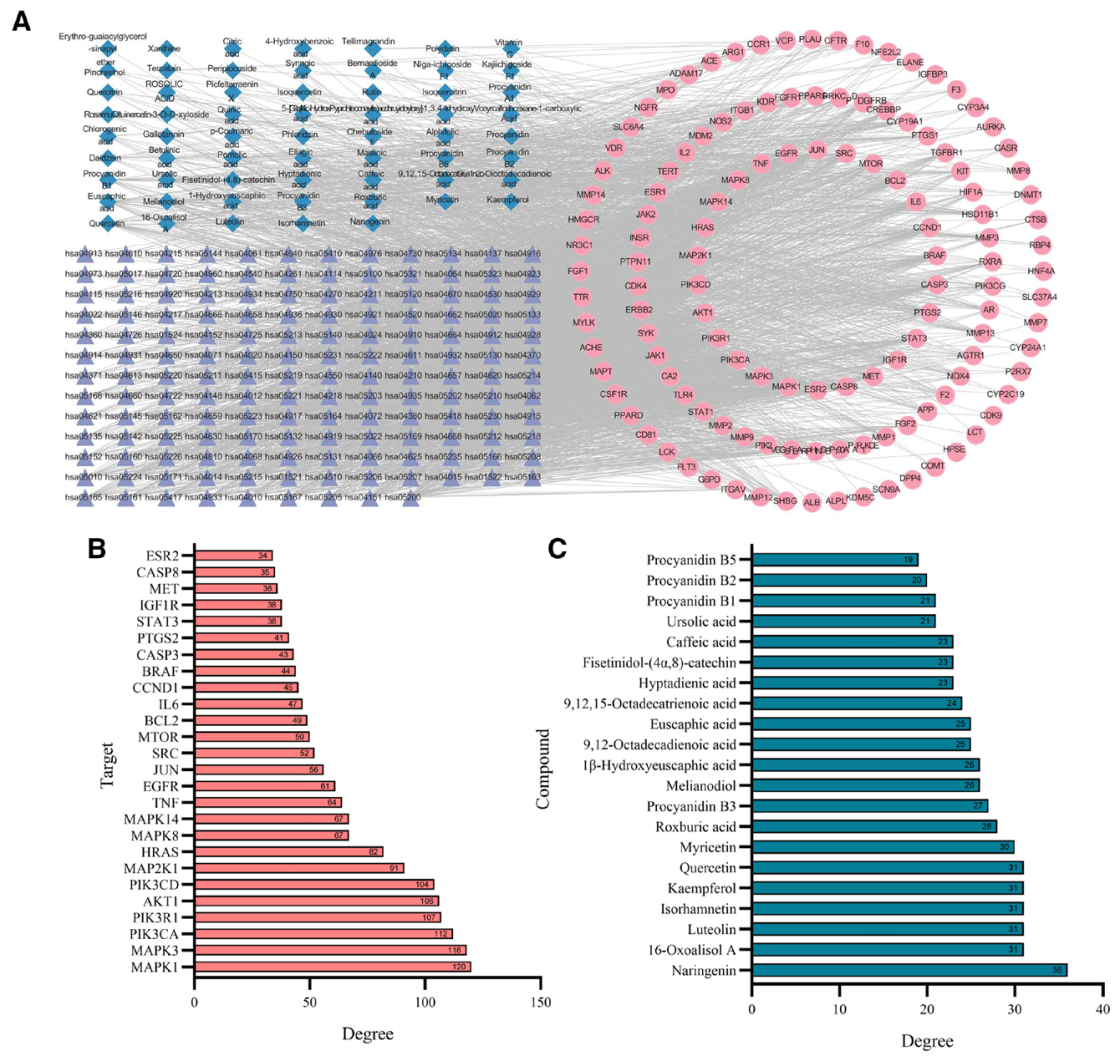
The key components and targets were identified by the compound-target-pathway network. (A) The compound-target-pathway network; 26 key targets (B) and 21 key components (C) were identified in the herb-compound-target network by selecting nodes with a degree ≥2-fold median. EGFR = epidermal growth factor receptor, PTGS2 = prostaglandin G/H synthase 2, SRC = proto-oncogene tyrosine-protein kinase Src, TNF = tumor necrosis factor.

The 125 common targets, 63 components of *R. roxburghii* Tratt., and 153 pathways were used to construct the compound-target-pathway network (Fig. 6A). We identified 26 key targets with a degree value >2-fold median. These targets include TNF, PTGS2, EGFR, SRC, mitogen-activated protein kinase 1, mitogen-activated protein kinase 3, phosphatidylinositol-4,5-bisphosphate 3-kinase catalytic subunit alpha, phosphoinositide-3-kinase regulatory subunit 1, AKT serine/threonine kinase 1, phosphatidylinositol-4,5-bisphosphate 3-kinase catalytic subunit delta, mitogen-activated protein kinase kinase 1, and others (Fig. 6B). In the same way, we also analyzed 21 key components in this network, including naringenin, 16-oxoalisol A, luteolin, isorhamnetin, kaempferol, quercetin, myricetin, roxburic acid, procyanidin B3, melianodiol, 1β-hydroxyeuscaphic acid, 9,12-octadecadienoic acid, and euscaphic acid (Fig. 6C).

### 3.6. Interactions between core components and core targets

**Figure 7.**
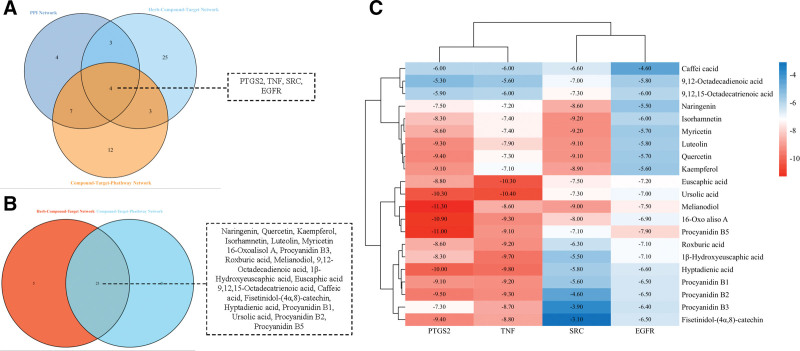
Interactions between core components and core targets. (A) The core targets based on PPI, herb-compound-target, and compound-target-pathway intersections; (B) the core components based on herb-compound-target and compound-target-pathway intersections; and (C) binding energy thermograms for core target and core component docking. EGFR = epidermal growth factor receptor, PPI = protein-protein interaction, PTGS2 = prostaglandin G/H synthase 2, SRC = proto-oncogene tyrosine-protein kinase Src, TNF = tumor necrosis factor.

We obtained the 4 common targets as the core targets of *R. roxburghii* Tratt. in fracture healing through the Venn diagram of the 3 networks: PPI, herb-compound-target, and compound-target-pathway(Fig. 7A). Then, we took the intersection based on the latter 2 networks to identify the 21 core components (Fig. 7B). The structure of 22 components was then uploaded to AMDock to analyze its potential for docking with TNF, PTGS2, EGFR, and SRC.

As shown in Figure 7C, the molecular docking results showed that most of the flavonoids had better affinity with PTGS2, followed by TNF. Except for the compounds (1β-hydroxy euscaphic acid, hyptadienic acid, procyanidin B1, procyanidin B2, procyanidin B3, and fisetinidol-(4α,8)-catechin), all the other compounds had better affinity with SRC. However, most of these compounds have lower affinity for EGFR than for the other 3 targets. However, overall, the flavonoids naringenin, quercetin, kaempferol, isorhamnetin, luteolin, and myricetin had better affinity for all 4 targets. In addition, melianodiol, ursolic acid, and procyanidin B5 were the highest affinity components for PTGS2, TNF, and EGFR, respectively, and isorhamnetin and myricetin were the active components with the highest affinity for SRC.

### 3.7. The results of molecular docking visualization

**Figure 8.**
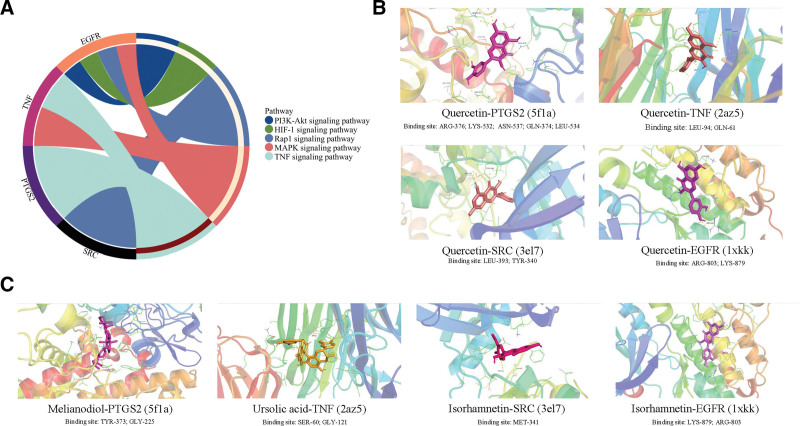
Molecular docking visualization and enrichment analysis circles. (A) Enrichment analysis circles; (B) the visualization images of quercetin docking with 4 core targets; and (C) the visualization images of the 4 core targets docked with the best affinity components. EGFR = epidermal growth factor receptor, HIF-1 = hypoxia-inducible factor 1, MAPK = mitogen-activated protein kinase, PI3K-Akt = phosphoinositide 3-kinase/protein kinase B, PTGS2 = prostaglandin G/H synthase 2, SRC = proto-oncogene tyrosine-protein kinase Src, TNF = tumor necrosis factor.

To further understand the role of core targets in fracture healing, we constructed enrichment analysis circles. As shown in Figure 8A, the core targets are involved in the following signaling pathways: SRC in the Rap1 signaling pathway; PTGS2 in the TNF signaling pathway; TNF in both the TNF signaling pathway and the MAPK signaling pathway; and EGFR in the PI3K-Akt signaling pathway, the HIF-1 signaling pathway, the Rap1 signaling pathway, and the MAPK signaling pathway.

We chose quercetin as a representative component of flavonoids to analyze its binding sites with the 4 targets. The results are shown in Figure 8B. The binding sites of quercetin with PTGS2 were ARG-376, LYS-532, ASN-537, GLN-374, and LEU-534. For TNF, the binding sites were LEU-94 and GLN-61. For SRC, the binding sites were LEU-393 and TYR-340. For EGFR, the binding sites were ARG-803 and LYS-879. Figure 8C depicts the binding sites of the components with the highest affinity for the 4 targets.

## 4. Discussion

In this study, we systematically analyzed the mechanism of *R. roxburghii* Tratt. in promoting fracture healing through integrated network pharmacology and molecular docking analysis. Our findings suggest that *R. roxburghii* Tratt. exerts its therapeutic effects by synergistically regulating osteogenesis and angiogenesis-related signaling pathways through bioactive components interacting with core targets.

Fracture healing is a multifaceted BP characterized by the orchestrated interplay of diverse cellular and molecular mechanisms, among which osteogenesis and angiogenesis emerge as critical drivers of bone regeneration.^[15]^ During the fracture healing, osteogenesis and angiogenesis are intricately coordinated through a network of molecular interactions.^[16]^ Cells within the fracture callus rely on angiogenesis for oxygen and nutrient supply, and during the fibrocartilaginous callus stage of fracture healing, angiogenesis-driven vessels form and promote bone formation by delivering progenitor cells to the site.^[17,18]^ Therefore, the increase in angiogenesis ability could contribute to bone healing. Angiogenesis, the formation of new blood vessels, is also crucial for fracture healing as it ensures the delivery of oxygen and nutrients to the healing site, facilitating tissue regeneration.^[19]^ In our research, GO enrichment analysis highlighted that the BPs influenced by *R. roxburghii* Tratt. include EGFR signaling pathway and vascular endothelial growth factor signaling pathway. VEGF is a key mediator of angiogenesis, and its upregulation enhances angiogenesis during fracture healing, thereby recruiting osteoblast progenitor cells to stimulate bone formation.^[20,21]^ However, the activation of EGFR signaling leads to decreased bone formation and enhanced bone resorption.^[22]^

Additionally, differentiation, immigration, and proliferation of fibroblasts and the inflammatory response are key processes of osteogenesis.^[23]^ Fibroblasts play a significant regulatory role in fracture healing by producing extracellular matrix components and growth factors that promote tissue repair.^[24]^ The fibroblast growth factor receptor signaling pathway, which was also enriched, plays a pivotal role in fibroblast proliferation and differentiation,^[25]^ further supporting the role of *R. roxburghii* Tratt. in promoting fracture healing. Our research revealed that *R. roxburghii* Tratt. is involved in several signaling pathways related to fracture healing, including the PI3K-Akt signaling pathway, MAPK signaling pathway, HIF-1 signaling pathway, TNF signaling pathway, and Rap1 signaling pathway. These pathways are known to regulate angiogenesis, fibroblast proliferation, and inflammatory response. For instance, TNF is a pleiotropic inflammatory cytokine that specifically promotes osteoclastogenesis in these common pathological bone diseases through multiple mechanisms.^[26]^ The PI3K-Akt and MAPK pathways are also essential for cell survival, proliferation, and migration,^[27]^ which, as a regulator during bone metabolism, can promote bone formation and differentiation of immature osteoblasts into mature osteoblasts.^[20,28,29]^ The HIF-1 signaling pathway has been demonstrated to play a critical role in maintaining bone homeostasis and promoting angiogenesis.^[30]^ Furthermore, the enhanced expression of HIF-1α has been shown to stimulate both angiogenic and osteogenic differentiation, thereby facilitating osteogenesis in bone marrow-derived mesenchymal stem cells.^[31]^

To further elucidate the core targets and components of *R. roxburghii* Tratt., we performed a molecular docking to identify 4 core targets – TNF, PTGS2, EGFR, and SRC – that are central to the fracture healing effects of *R. roxburghii* Tratt. These targets are involved in multiple signaling pathways that regulate inflammation, angiogenesis, and cell proliferation, all of which are critical for bone repair. The molecular docking results revealed that flavonoids such as quercetin, kaempferol, and isorhamnetin exhibit strong binding affinity for these core targets, suggesting their potential to modulate key pathways involved in fracture healing. For example, quercetin showed high affinity for PTGS2, TNF, SRC, and EGFR, indicating its potential to regulate inflammation, angiogenesis, and cell proliferation. The binding sites of quercetin with these targets were identified, providing insights into the molecular interactions that may underlie the therapeutic effects of *R. roxburghii* Tratt. in fracture healing. Specifically, quercetin’s interaction with PTGS2 at ARG-376 and LYS-532 suggests that it may inhibit PTGS2 activity, thereby reducing inflammation and pain at the fracture site. Similarly, the high affinity of isorhamnetin and myricetin for SRC indicates that these compounds may enhance cell migration and tissue regeneration by modulating SRC signaling. The binding of melianodiol and procyanidin B5 to EGFR further supports their potential to promote angiogenesis and fibroblast proliferation, which are essential for bone repair. These findings are consistent with previous studies that have shown the anti-inflammatory and pro-angiogenic properties of these flavonoids.

In summary, our study reveals that *R. roxburghii* Tratt. enhances fracture healing by regulating key signaling pathways such as PI3K-Akt, MAPK, HIF-1, TNF, and Rap1, which are crucial for angiogenesis and osteogenesis. Molecular docking analysis indicates that bioactive components such as quercetin, kaempferol, and isorhamnetin strongly bind to core targets (TNF, PTGS2, EGFR, and SRC), suggesting their potential to influence critical pathways involved in bone repair. These findings provide a foundation for further exploration of *R. roxburghii* Tratt. as a therapeutic agent for fracture healing, highlighting its promising role in future clinical applications.

## 5. Conclusion

In conclusion, our study demonstrates that *R. roxburghii* Tratt. enhances fracture healing through multitarget, components, and pathway regulation of osteogenesis and angiogenesis. Through integrated network pharmacology and molecular docking analysis, we identified bioactive components such as quercetin, kaempferol, and isorhamnetin, which exhibit strong binding affinity to core targets (TNF, PTGS2, EGFR, and SRC). These components regulate inflammation, angiogenesis, and cell proliferation-related pathways, thereby promoting bone repair. These findings suggest that *R. roxburghii* Tratt. has significant potential as a therapeutic agent for fracture healing, and further experimental validation is warranted to confirm these results.

## Author contributions

**Conceptualization:** Qifeng Ying, Jun Zhang, Zixue Xuan.

**Methodology:** Pingping Luo.

**Data curation:** Zhongxiu Wu, Jiongnan Xu.

**Investigation:** Hongshi Liu.

**Writing – original draft:** Zhongxiu Wu.

## Supplementary Material

## References

[1] Huang J Huang J Li N Wang L Xiao Q. FBN2 promotes the proliferation, mineralization, and differentiation of osteoblasts to accelerate fracture healing. Sci Rep. 2025;15:4843. 39924543 10.1038/s41598-025-89215-6 PMC11808093

[2] Burgan J Rahmati M Lee M Saiz AM. Innate immune response to bone fracture healing. Bone. 2025;190:117327. 39522707 10.1016/j.bone.2024.117327

[3] Łuczak JW Palusińska M Matak D. The future of bone repair: emerging technologies and biomaterials in bone regeneration. Int J Mol Sci. 2024;25:12766. 39684476 10.3390/ijms252312766 PMC11641768

[4] Hu Y Cen M Hu Y. Exploring the role of OIP5-AS1 in the mechanisms of delayed fracture healing: functional insights and clinical implications. J Orthop Surg Res. 2025;20:32. 39794811 10.1186/s13018-024-05428-x PMC11724555

[5] Wu X Zhou X Liang S Zhu X Dong Z. The mechanism of pyrroloquinoline quinone influencing the fracture healing process of estrogen-deficient mice by inhibiting oxidative stress. Biomed Pharmacother. 2021;139:111598. 33895522 10.1016/j.biopha.2021.111598

[6] Gharu E John B. Nonunion of fractures: a review of epidemiology, diagnosis, and clinical features in recent literature. Ind J Orthop. 2024;58:1680–5. 10.1007/s43465-024-01249-6 PMC11628462 39664347

[7] Bowers KM Anderson DE. Delayed union and nonunion: current concepts, prevention, and correction: a review. Bioengineering (Basel). 2024;11:525. 38927761 10.3390/bioengineering11060525 PMC11201148

[8] Zhang J Shen W Liu F He H Han S Luo L. Fracture-healing effects of Rhizoma Musae ethanolic extract: an integrated study using UHPLC-Q-Exactive-MS/MS, network pharmacology, and molecular docking. PLoS One. 2025;20:e0313743. 39808649 10.1371/journal.pone.0313743 PMC11731732

[9] Li X Ling Y Huang X. *Rosa roxburghii* Tratt Fruit extract prevents Dss-induced ulcerative colitis in mice by modulating the gut microbiota and the IL-17 signaling pathway. Nutrients. 2023;15:4560. 37960213 10.3390/nu15214560 PMC10650662

[10] Jain A Sarsaiya S Gong Q Wu Q Shi J. Chemical diversity, traditional uses, and bioactivities of *Rosa roxburghii* Tratt: a comprehensive review. Pharmacol Ther. 2024;259:108657. 38735487 10.1016/j.pharmthera.2024.108657

[11] Chunxue F Yulin D Yaxi Z Nian X. Research progress on pharmacological activity of *Rosa roxburghii* Tratt. Life Sci Instruments. 2021;19:14–21.

[12] Yuan H Wang Y Chen H Cai X. Protective effect of flavonoids from *Rosa roxburghii* Tratt on myocardial cells via autophagy. 3 Biotech. 2020;10:58. 10.1007/s13205-019-2049-1 PMC6976074 32015954

[13] Zhang P Zhang D Zhou W. Network pharmacology: towards the artificial intelligence-based precision traditional Chinese medicine. Brief Bioinform. 2023;25:bbad518. 38197310 10.1093/bib/bbad518 PMC10777171

[14] Nogales C Mamdouh ZM List M Kiel C Casas AI Schmidt HH. Network pharmacology: curing causal mechanisms instead of treating symptoms. Trends Pharmacol Sci. 2022;43:136–50. 34895945 10.1016/j.tips.2021.11.004

[15] Einhorn TA Gerstenfeld LC. Fracture healing: mechanisms and interventions. Nat Rev Rheumatol. 2015;11:45–54. 25266456 10.1038/nrrheum.2014.164 PMC4464690

[16] Bixel MG Sivaraj KK Timmen M. Angiogenesis is uncoupled from osteogenesis during calvarial bone regeneration. Nat Commun. 2024;15:4575. 38834586 10.1038/s41467-024-48579-5 PMC11150404

[17] Liu Y Fang J Zhang Q. Wnt10b-overexpressing umbilical cord mesenchymal stem cells promote critical size rat calvarial defect healing by enhanced osteogenesis and VEGF-mediated angiogenesis. J Orthop Transl. 2020;23:29–37. 10.1016/j.jot.2020.02.009 PMC7248289 32477867

[18] Zhang S Chen L Zhang C. Osteoking exerts pro‑osteogenic and anti‑adipogenic effects in promoting bone fracture healing via EGF/EGFR/HDAC1/Wnt/β‑catenin signaling. Int J Mol Med. 2025;55:75. 40052602 10.3892/ijmm.2025.5516 PMC11936482

[19] Ali S Singh A Mahdi AA Srivastava RN. CYR61-an angiogenic biomarker to early predict the impaired healing in diaphyseal tibial fractures. J Orthop Transl. 2017;10:5–11. 10.1016/j.jot.2017.02.004 PMC5822955 29662755

[20] Wei X Wang J Deng YY. Tubiechong patching promotes tibia fracture healing in rats by regulating angiogenesis through the VEGF/ERK1/2 signaling pathway. J Ethnopharmacol. 2023;301:115851. 36273748 10.1016/j.jep.2022.115851

[21] Song N Zhao Z Ma X. Naringin promotes fracture healing through stimulation of angiogenesis by regulating the VEGF/VEGFR-2 signaling pathway in osteoporotic rats. Chem Biol Interact. 2017;261:11–7. 27833010 10.1016/j.cbi.2016.10.020

[22] Lees-Shepard JB Flint K Fisher M. Cross-talk between EGFR and BMP signals regulates chondrocyte maturation during endochondral ossification. Dev Dyn. 2022;251:75–94. 34773433 10.1002/dvdy.438

[23] Wang H Qi LL Shema C. Advances in the role and mechanism of fibroblasts in fracture healing. Front Endocrinol. 2024;15:1350958. 10.3389/fendo.2024.1350958 PMC10925620 38469138

[24] Meng GL Hu YY Pu Q. Reciprocal action between BMP-2 and BMP-3 in cultured fibroblast in vitro. Chin J Traumatol. 2003;6:3–7. 12542956

[25] Hankenson KD Gagne K Shaughnessy M. Extracellular signaling molecules to promote fracture healing and bone regeneration. Adv Drug Deliv Rev. 2015;94:3–12. 26428617 10.1016/j.addr.2015.09.008

[26] Zhao B. Intrinsic restriction of TNF-mediated inflammatory osteoclastogenesis and bone resorption. Front Endocrinol. 2020;11:583561. 10.3389/fendo.2020.583561 PMC7578415 33133025

[27] Sun Y Liu WZ Liu T Feng X Yang N Zhou HF. Signaling pathway of MAPK/ERK in cell proliferation, differentiation, migration, senescence and apoptosis. J Recept Signal Transduct Res. 2015;35:600–4. 26096166 10.3109/10799893.2015.1030412

[28] Liu Y Liu J Xia T. MiR-21 promotes fracture healing by activating the PI3K/Akt signaling pathway. Eur Rev Med Pharmacol Sci. 2019;23:2727–33. 31002122 10.26355/eurrev_201904_17544

[29] Chen H Fang C Zhi X. Neobavaisoflavone inhibits osteoclastogenesis through blocking RANKL signalling-mediated TRAF6 and c-Src recruitment and NF-κB, MAPK and Akt pathways. J Cell Mol Med. 2020;24:9067–84. 32604472 10.1111/jcmm.15543 PMC7417698

[30] Ke Q Costa M. Hypoxia-inducible factor-1 (HIF-1). Mol Pharmacol. 2006;70:1469–80. 16887934 10.1124/mol.106.027029

[31] Song S Zhang G Chen X. HIF-1α increases the osteogenic capacity of ADSCs by coupling angiogenesis and osteogenesis via the HIF-1α/VEGF/AKT/mTOR signaling pathway. J Nanobiotechnol. 2023;21:257. 10.1186/s12951-023-02020-z PMC10405507 37550736

