# Supplementary material for: Rosa roxburghii Tratt. enhances fracture healing through multitarget regulation of osteogenesis and angiogenesis: Integrated network pharmacology and molecular docking analysis
Source: Medicine (Baltimore). 2026 Mar 13;105(11):e48071. doi: 10.1097/MD.0000000000048071 (PMC12991575; doi:10.1097/MD.0000000000048071)
Supplement: Supplementary file 1 [file medi-105-e48071-s001.docx]

**Tab. S1 Corresponding 548 targets of the 64 components of *Rosa roxburghii* Tratt.**

| NO. | Target | NO. | Target | NO. | Target | NO. | Target | NO. | Target |
| --- | --- | --- | --- | --- | --- | --- | --- | --- | --- |
| 1 | PTGS1 | 111 | TNKS2 | 221 | KDM5C | 331 | ADAMTS5 | 441 | CLK2 |
| 2 | PTGS2 | 112 | TNKS | 222 | KDM4A | 332 | ITGAV | 442 | CLK3 |
| 3 | GABRA1 | 113 | TOP1 | 223 | KDM4C | 333 | CCKBR | 443 | CDK3 |
| 4 | LCT | 114 | TERT | 224 | KDM6B | 334 | TYMS | 444 | CDK16 |
| 5 | GRIA2 | 115 | NQO2 | 225 | MMP8 | 335 | MMEL1 | 445 | MAPK15 |
| 6 | GSK3B | 116 | ADRA2C | 226 | PTGDR | 336 | CASP2 | 446 | CDK18 |
| 7 | SQLE | 117 | RPS6KA3 | 227 | TUBB1 | 337 | JUN | 447 | MAPK7 |
| 8 | F10 | 118 | NMUR2 | 228 | KMO | 338 | VDR | 448 | CDK17 |
| 9 | BACE1 | 119 | ADRA2A | 229 | ACE | 339 | GLI1 | 449 | MAPKAPK2 |
| 10 | VCP | 120 | TNF | 230 | ITGAL | 340 | PTPA | 450 | RBP4 |
| 11 | TNNC1 | 121 | IL2 | 231 | EGLN3 | 341 | TYRO3 | 451 | HDAC2 |
| 12 | SERPINE1 | 122 | PDE5A | 232 | CPA3 | 342 | PRKCG | 452 | JAK1 |
| 13 | PTPN1 | 123 | SLC29A1 | 233 | NOTUM | 343 | PRKCE | 453 | PIK3CD |
| 14 | PTPN2 | 124 | ESR1 | 234 | GRK2 | 344 | ADORA3 | 454 | ROCK2 |
| 15 | AMY1A | 125 | CFTR | 235 | TAS1R3 | 345 | TAS2R31 | 455 | PIM3 |
| 16 | BCL2 | 126 | PFKFB3 | 236 | POLA1 | 346 | GRM5 | 456 | LIMK2 |
| 17 | NOX4 | 127 | GRK6 | 237 | POLB | 347 | CES1 | 457 | MAPK3 |
| 18 | AVPR2 | 128 | MCL1 | 238 | RNPEP | 348 | SLC5A2 | 458 | CHRM2 |
| 19 | AKR1B1 | 129 | PLG | 239 | KDM4B | 349 | RXRA | 459 | GRIK1 |
| 20 | XDH | 130 | AR | 240 | KDM5B | 350 | HSD17B14 | 460 | GRIK2 |
| 21 | MAOA | 131 | ALDH2 | 241 | KDM4D | 351 | CTSB | 461 | NR3C2 |
| 22 | IGF1R | 132 | TBXAS1 | 242 | ECE1 | 352 | CLK1 | 462 | NR1I3 |
| 23 | FLT3 | 133 | MGAM | 243 | AMPD3 | 353 | DYRK1B | 463 | CNR2 |
| 24 | CYP19A1 | 134 | HTR2A | 244 | PTPN22 | 354 | IGFBP3 | 464 | FKBP1A |
| 25 | EGFR | 135 | HTR2C | 245 | KIT | 355 | YWHAG | 465 | BRD4 |
| 26 | F2 | 136 | ESRRB | 246 | FGFR1 | 356 | SIRT2 | 466 | SORD |
| 27 | CA2 | 137 | MIF | 247 | DNM1 | 357 | CCNE1 | 467 | CCNC |
| 28 | PIM1 | 138 | CBR1 | 248 | PLA2G2A | 358 | CDK4 | 468 | CDK8 |
| 29 | ALOX5 | 139 | SLC6A2 | 249 | PLA2G5 | 359 | HNF4A | 469 | CREBBP |
| 30 | AURKB | 140 | MAOB | 250 | PLA2G10 | 360 | ST6GAL1 | 470 | LRRK2 |
| 31 | DRD4 | 141 | PON1 | 251 | KLK1 | 361 | SLC5A4 | 471 | MAPK11 |
| 32 | ADORA1 | 142 | STS | 252 | KLK2 | 362 | SLC5A1 | 472 | SCN9A |
| 33 | CA7 | 143 | TLR9 | 253 | HIF1A | 363 | SLC28A3 | 473 | P2RX7 |
| 34 | GLO1 | 144 | PPARA | 254 | KCNH2 | 364 | HRAS | 474 | MERTK |
| 35 | MPO | 145 | PLAT | 255 | FLT1 | 365 | ADORA2B | 475 | PGGT1B |
| 36 | PIK3R1 | 146 | PLAU | 256 | CHEK1 | 366 | MMP7 | 476 | DRD2 |
| 37 | ADORA2A | 147 | CA5B | 257 | WEE1 | 367 | ADAM17 | 477 | PDE2A |
| 38 | DAPK1 | 148 | PPARG | 258 | LNPEP | 368 | PNP | 478 | PDE4B |
| 39 | PYGL | 149 | DYRK1A | 259 | POLK | 369 | GBA | 479 | SLC6A3 |
| 40 | CA1 | 150 | MAPK14 | 260 | HSP90AA1 | 370 | SLC28A2 | 480 | CCNE2 |
| 41 | SRC | 151 | PGD | 261 | LGALS3 | 371 | ADK | 481 | PAK1 |
| 42 | PTK2 | 152 | ST3GAL3 | 262 | LGALS9 | 372 | TRPA1 | 482 | CHRM1 |
| 43 | HSD17B2 | 153 | FUT7 | 263 | FGF1 | 373 | MAPK9 | 483 | PSEN2 |
| 44 | KDR | 154 | FUT4 | 264 | FGF2 | 374 | SOAT1 | 484 | PDE10A |
| 45 | MMP13 | 155 | STAT1 | 265 | HPSE | 375 | SOAT2 | 485 | ALPL |
| 46 | MMP3 | 156 | MMP14 | 266 | PTAFR | 376 | OPRM1 | 486 | MAPK8 |
| 47 | CA3 | 157 | PGF | 267 | PTGER2 | 377 | OGA | 487 | MAPK10 |
| 48 | ALOX15 | 158 | VEGFA | 268 | PTGER1 | 378 | FBP1 | 488 | IRAK4 |
| 49 | ABCC1 | 159 | EIF4A1 | 269 | PTGER4 | 379 | FTO | 489 | HTR2B |
| 50 | PLK1 | 160 | DNMT1 | 270 | SERPINA6 | 380 | NGFR | 490 | ADRA2B |
| 51 | CA6 | 161 | LDHA | 271 | SHBG | 381 | NR1H4 | 491 | DRD1 |
| 52 | CDK1 | 162 | LDHB | 272 | G6PD | 382 | ACLY | 492 | DRD3 |
| 53 | MMP9 | 163 | COMT | 273 | CD81 | 383 | CDC25A | 493 | CYP2D6 |
| 54 | CA12 | 164 | BCL2L1 | 274 | PTGIR | 384 | FABP4 | 494 | HTR6 |
| 55 | MMP2 | 165 | ALB | 275 | SLC6A4 | 385 | FABP3 | 495 | HTR1B |
| 56 | PKN1 | 166 | TPMT | 276 | FNTA | 386 | FABP5 | 496 | ATP1A1 |
| 57 | CA14 | 167 | ERBB2 | 277 | NR3C1 | 387 | FFAR1 | 497 | PRKCQ |
| 58 | CA9 | 168 | CCND1 | 278 | PTPRF | 388 | FABP2 | 498 | PRUNE1 |
| 59 | CSNK2A1 | 169 | PDGFRB | 279 | PPARD | 389 | HSD11B2 | 499 | PDE11A |
| 60 | ALOX12 | 170 | FLT4 | 280 | PTGER3 | 390 | HAO1 | 500 | MAP3K11 |
| 61 | MET | 171 | PLK4 | 281 | NOS2 | 391 | UGT2B7 | 501 | MAP3K9 |
| 62 | CA4 | 172 | TEK | 282 | CASP3 | 392 | GABBR1 | 502 | P2RY12 |
| 63 | NEK2 | 173 | AURKA | 283 | CASP8 | 393 | SLC22A6 | 503 | MKNK2 |
| 64 | CXCR1 | 174 | MAP3K8 | 284 | FDFT1 | 394 | GABRA2 | 504 | F7 |
| 65 | CAMK2B | 175 | BRAF | 285 | RORC | 395 | S1PR2 | 505 | NR1H2 |
| 66 | ALK | 176 | EPHB4 | 286 | ACP1 | 396 | P2RX3 | 506 | IKBKE |
| 67 | AKT1 | 177 | HSPA1A | 287 | CDC25B | 397 | DLG4 | 507 | TBK1 |
| 68 | ABCB1 | 178 | FGR | 288 | HMGCR | 398 | TRPM8 | 508 | BCL2A1 |
| 69 | NEK6 | 179 | LYN | 289 | CYP17A1 | 399 | SCD | 509 | F2RL1 |
| 70 | PLA2G1B | 180 | GSR | 290 | PTGFR | 400 | FAAH | 510 | GLRA1 |
| 71 | CA5A | 181 | DAO | 291 | CCR1 | 401 | IDO1 | 511 | GLRA2 |
| 72 | CYP1B1 | 182 | HSD17B3 | 292 | LTB4R | 402 | ALOX5AP | 512 | TTL |
| 73 | AXL | 183 | CES2 | 293 | CASP1 | 403 | DPP4 | 513 | ATP12A |
| 74 | ABCG2 | 184 | SNCA | 294 | EDNRB | 404 | PTPN11 | 514 | PRKCB |
| 75 | NUAK1 | 185 | SRD5A2 | 295 | ITGB1 | 405 | CNR1 | 515 | RPS6KA5 |
| 76 | AKR1C2 | 186 | HDAC6 | 296 | PTPN6 | 406 | SREBF2 | 516 | EGLN1 |
| 77 | AKR1C1 | 187 | HDAC8 | 297 | NPC1L1 | 407 | SLC10A1 | 517 | TRPV1 |
| 78 | AKR1C3 | 188 | ERN1 | 298 | RORA | 408 | SLC10A2 | 518 | FFAR4 |
| 79 | AKR1C4 | 189 | MMP1 | 299 | IMPDH1 | 409 | SAE1 | 519 | SLC16A1 |
| 80 | CA13 | 190 | HCAR2 | 300 | IMPDH2 | 410 | CDC25C | 520 | IL6 |
| 81 | AKR1A1 | 191 | TLR4 | 301 | FABP1 | 411 | SMO | 521 | GLUL |
| 82 | GPR35 | 192 | MAPK1 | 302 | PPP2CA | 412 | HCRTR2 | 522 | ENPP2 |
| 83 | MAPT | 193 | FYN | 303 | PTGES | 413 | HCRTR1 | 523 | PLA2G4A |
| 84 | KDM4E | 194 | LCK | 304 | CASP7 | 414 | ACACB | 524 | CYP26B1 |
| 85 | TOP2A | 195 | PIK3CB | 305 | PTGDR2 | 415 | CSF1R | 525 | CYP26A1 |
| 86 | INSR | 196 | CYP1A2 | 306 | PGR | 416 | MDM2 | 526 | RXRB |
| 87 | ACHE | 197 | CYP2C9 | 307 | CASR | 417 | MAP3K14 | 527 | RORB |
| 88 | MYLK | 198 | CYP3A4 | 308 | HSP90AB1 | 418 | JAK2 | 528 | CMA1 |
| 89 | SYK | 199 | CYP2C19 | 309 | MME | 419 | DUT | 529 | CTSG |
| 90 | PIK3CG | 200 | PIK3CA | 310 | REN | 420 | CCNT1 | 530 | OXER1 |
| 91 | APEX1 | 201 | ELANE | 311 | NR1H3 | 421 | GYS1 | 531 | GRM2 |
| 92 | PTPRS | 202 | F3 | 312 | THRA | 422 | MTOR | 532 | DAGLA |
| 93 | ESR2 | 203 | HSD11B1 | 313 | THRB | 423 | ABL1 | 533 | ABHD6 |
| 94 | MPG | 204 | NFE2L2 | 314 | PRKCH | 424 | PDE3A | 534 | PRKAG1 |
| 95 | SLC22A12 | 205 | STAT3 | 315 | SIGMAR1 | 425 | PDE3B | 535 | GDA |
| 96 | CDK5R1 | 206 | TDP1 | 316 | AGTR1 | 426 | S1PR3 | 536 | BRD2 |
| 97 | CCNB3 | 207 | SRD5A1 | 317 | PTGIS | 427 | S1PR1 | 537 | BRDT |
| 98 | ARG1 | 208 | EPHX2 | 318 | CTSA | 428 | NAMPT | 538 | BRD3 |
| 99 | CDK6 | 209 | SLC37A4 | 319 | CYP51A1 | 429 | CHUK | 539 | CHIA |
| 100 | CDK2 | 210 | PRKCD | 320 | AMPD2 | 430 | TGFBR1 | 540 | CYP24A1 |
| 101 | TYR | 211 | PRKCA | 321 | HDAC1 | 431 | AURKAIP1 | 541 | ROCK1 |
| 102 | HSD17B1 | 212 | PDE4D | 322 | PDE6D | 432 | MAP2K1 | 542 | PRKACA |
| 103 | AHR | 213 | PDE9A | 323 | BCHE | 433 | F9 | 543 | CAPN1 |
| 104 | ESRRA | 214 | PDE1B | 324 | ITGA4 | 434 | GSK3A | 544 | RXRG |
| 105 | APP | 215 | EDNRA | 325 | ANPEP | 435 | CDK7 | 545 | DNTT |
| 106 | PARP1 | 216 | NEU4 | 326 | GRB2 | 436 | CDK9 | 546 | CYSLTR1 |
| 107 | TTR | 217 | CHRNA4 | 327 | ITGA2B | 437 | STK16 | 547 | PLEC |
| 108 | MMP12 | 218 | CHRNA7 | 328 | PREP | 438 | TTK | 548 | AGTR2 |
| 109 | CD38 | 219 | KDM3A | 329 | ITGB7 | 439 | CDK5 |  |  |
| 110 | AKR1B10 | 220 | KDM2A | 330 | GPBAR1 | 440 | CLK4 |  |  |

**Tab. S2 153 signaling pathways of KEGG analysis**

| Term | Pathway | Term | Pathway | Term | Pathway |
| --- | --- | --- | --- | --- | --- |
| hsa05200 | Pathways in cancer | hsa04620 | Toll-like receptor signaling pathway | hsa04929 | GnRH secretion |
| hsa05205 | Proteoglycans in cancer | hsa05166 | Human T-cell leukemia virus 1 infection | hsa04910 | Insulin signaling pathway |
| hsa04933 | AGE-RAGE signaling pathway in diabetic complications | hsa05220 | Chronic myeloid leukemia | hsa04726 | Serotonergic synapse |
| hsa01522 | Endocrine resistance | hsa04657 | IL-17 signaling pathway | hsa05130 | Pathogenic Escherichia coli infection |
| hsa04151 | PI3K-Akt signaling pathway | hsa05152 | Tuberculosis | hsa04658 | Th1 and Th2 cell differentiation |
| hsa01521 | EGFR tyrosine kinase inhibitor resistance | hsa05162 | Measles | hsa05120 | Epithelial cell signaling in Helicobacter pylori infection |
| hsa05215 | Prostate cancer | hsa04722 | Neurotrophin signaling pathway | hsa04666 | Fc gamma R-mediated phagocytosis |
| hsa05167 | Kaposi sarcoma-associated herpesvirus infection | hsa04660 | T cell receptor signaling pathway | hsa05146 | Amoebiasis |
| hsa05161 | Hepatitis B | hsa04630 | JAK-STAT signaling pathway | hsa04213 | Longevity regulating pathway - multiple species |
| hsa05235 | PD-L1 expression and PD-1 checkpoint pathway in cancer | hsa05225 | Hepatocellular carcinoma | hsa04211 | Longevity regulating pathway |
| hsa05218 | Melanoma | hsa04810 | Regulation of actin cytoskeleton | hsa04920 | Adipocytokine signaling pathway |
| hsa04625 | C-type lectin receptor signaling pathway | hsa05169 | Epstein-Barr virus infection | hsa04921 | Oxytocin signaling pathway |
| hsa05212 | Pancreatic cancer | hsa05213 | Endometrial cancer | hsa04115 | p53 signaling pathway |
| hsa05417 | Lipid and atherosclerosis | hsa05222 | Small cell lung cancer | hsa05022 | Pathways of neurodegeneration - multiple diseases |
| hsa04066 | HIF-1 signaling pathway | hsa04218 | Cellular senescence | hsa04923 | Regulation of lipolysis in adipocytes |
| hsa05230 | Central carbon metabolism in cancer | hsa04930 | Type II diabetes mellitus | hsa04936 | Alcoholic liver disease |
| hsa05219 | Bladder cancer | hsa05231 | Choline metabolism in cancer | hsa04960 | Aldosterone-regulated sodium reabsorption |
| hsa04510 | Focal adhesion | hsa05131 | Shigellosis | hsa04020 | Calcium signaling pathway |
| hsa05163 | Human cytomegalovirus infection | hsa05164 | Influenza A | hsa04670 | Leukocyte transendothelial migration |
| hsa05221 | Acute myeloid leukemia | hsa04664 | Fc epsilon RI signaling pathway | hsa04360 | Axon guidance |
| hsa05224 | Breast cancer | hsa04148 | Efferocytosis | hsa04024 | cAMP signaling pathway |
| hsa04015 | Rap1 signaling pathway | hsa04210 | Apoptosis | hsa05321 | Inflammatory bowel disease |
| hsa04917 | Prolactin signaling pathway | hsa05170 | Human immunodeficiency virus 1 infection | hsa04217 | Necroptosis |
| hsa05207 | Chemical carcinogenesis - receptor activation | hsa04621 | NOD-like receptor signaling pathway | hsa04750 | Inflammatory mediator regulation of TRP channels |
| hsa05223 | Non-small cell lung cancer | hsa04062 | Chemokine signaling pathway | hsa05100 | Bacterial invasion of epithelial cells |
| hsa04010 | MAPK signaling pathway | hsa05140 | Leishmaniasis | hsa04973 | Carbohydrate digestion and absorption |
| hsa04926 | Relaxin signaling pathway | hsa04550 | Signaling pathways regulating pluripotency of stem cells | hsa04215 | Apoptosis - multiple species |
| hsa04068 | FoxO signaling pathway | hsa05202 | Transcriptional misregulation in cancer | hsa05134 | Legionellosis |
| hsa04668 | TNF signaling pathway | hsa04611 | Platelet activation | hsa04730 | Long-term depression |
| hsa05142 | Chagas disease | hsa05203 | Viral carcinogenesis | hsa04540 | Gap junction |
| hsa05210 | Colorectal cancer | hsa04931 | Insulin resistance | hsa05323 | Rheumatoid arthritis |
| hsa05214 | Glioma | hsa04914 | Progesterone-mediated oocyte maturation | hsa04022 | cGMP-PKG signaling pathway |
| hsa04014 | Ras signaling pathway | hsa04928 | Parathyroid hormone synthesis, secretion and action | hsa04720 | Long-term potentiation |
| hsa05226 | Gastric cancer | hsa04912 | GnRH signaling pathway | hsa04530 | Tight junction |
| hsa05160 | Hepatitis C | hsa05132 | Salmonella infection | hsa04270 | Vascular smooth muscle contraction |
| hsa05211 | Renal cell carcinoma | hsa04371 | Apelin signaling pathway | hsa04064 | NF-kappa B signaling pathway |
| hsa04012 | ErbB signaling pathway | hsa01524 | Platinum drug resistance | hsa05144 | Malaria |
| hsa04919 | Thyroid hormone signaling pathway | hsa05010 | Alzheimer disease | hsa05020 | Prion disease |
| hsa05171 | Coronavirus disease - COVID-19 | hsa04140 | Autophagy - animal | hsa05168 | Herpes simplex virus 1 infection |
| hsa04370 | VEGF signaling pathway | hsa05133 | Pertussis | hsa04934 | Cushing syndrome |
| hsa04659 | Th17 cell differentiation | hsa04071 | Sphingolipid signaling pathway | hsa04114 | Oocyte meiosis |
| hsa05145 | Toxoplasmosis | hsa04932 | Non-alcoholic fatty liver disease | hsa04916 | Melanogenesis |
| hsa05165 | Human papillomavirus infection | hsa04650 | Natural killer cell mediated cytotoxicity | hsa04137 | Mitophagy - animal |
| hsa05208 | Chemical carcinogenesis - reactive oxygen species | hsa04662 | B cell receptor signaling pathway | hsa04261 | Adrenergic signaling in cardiomyocytes |
| hsa05206 | MicroRNAs in cancer | hsa04725 | Cholinergic synapse | hsa04610 | Complement and coagulation cascades |
| hsa05135 | Yersinia infection | hsa04520 | Adherens junction | hsa04913 | Ovarian steroidogenesis |
| hsa04915 | Estrogen signaling pathway | hsa05216 | Thyroid cancer | hsa04976 | Bile secretion |
| hsa05418 | Fluid shear stress and atherosclerosis | hsa05415 | Diabetic cardiomyopathy | hsa05410 | Hypertrophic cardiomyopathy |
| hsa04935 | Growth hormone synthesis, secretion and action | hsa04152 | AMPK signaling pathway | hsa05017 | Spinocerebellar ataxia |
| hsa04380 | Osteoclast differentiation | hsa04150 | mTOR signaling pathway | hsa04640 | Hematopoietic cell lineage |
| hsa04072 | Phospholipase D signaling pathway | hsa04613 | Neutrophil extracellular trap formation | hsa04061 | Viral protein interaction with cytokine and cytokine receptor |

**Tab. S3 491 biological process (BP) of GO analysis**

| Term | Biological process | Term | Biological process | Term | Biological process |
| --- | --- | --- | --- | --- | --- |
| GO:0051897 | positive regulation of phosphatidylinositol 3-kinase/protein kinase B signal transduction | GO:0048511 | rhythmic process | GO:0046854 | phosphatidylinositol phosphate biosynthetic process |
| GO:0030335 | positive regulation of cell migration | GO:0035094 | response to nicotine | GO:0034446 | substrate adhesion-dependent cell spreading |
| GO:0048009 | insulin-like growth factor receptor signaling pathway | GO:0009636 | response to toxic substance | GO:0051146 | striated muscle cell differentiation |
| GO:0043410 | positive regulation of MAPK cascade | GO:0032729 | positive regulation of type II interferon production | GO:0022408 | negative regulation of cell-cell adhesion |
| GO:0048013 | ephrin receptor signaling pathway | GO:0051726 | regulation of cell cycle | GO:0032956 | regulation of actin cytoskeleton organization |
| GO:0008286 | insulin receptor signaling pathway | GO:0030194 | positive regulation of blood coagulation | GO:0031648 | protein destabilization |
| GO:0007173 | epidermal growth factor receptor signaling pathway | GO:0046427 | positive regulation of receptor signaling pathway via JAK-STAT | GO:0032091 | negative regulation of protein binding |
| GO:0010628 | positive regulation of gene expression | GO:0006366 | transcription by RNA polymerase II | GO:0048863 | stem cell differentiation |
| GO:0038084 | vascular endothelial growth factor signaling pathway | GO:0042981 | regulation of apoptotic process | GO:0035162 | embryonic hemopoiesis |
| GO:0008543 | fibroblast growth factor receptor signaling pathway | GO:0048870 | cell motility | GO:0048266 | behavioral response to pain |
| GO:0018108 | peptidyl-tyrosine phosphorylation | GO:0048143 | astrocyte activation | GO:0097242 | amyloid-beta clearance |
| GO:0008284 | positive regulation of cell population proliferation | GO:0060749 | mammary gland alveolus development | GO:1904385 | cellular response to angiotensin |
| GO:0043406 | positive regulation of MAP kinase activity | GO:0050731 | positive regulation of peptidyl-tyrosine phosphorylation | GO:0006606 | protein import into nucleus |
| GO:0048661 | positive regulation of smooth muscle cell proliferation | GO:0007155 | cell adhesion | GO:0006874 | intracellular calcium ion homeostasis |
| GO:0001934 | positive regulation of protein phosphorylation | GO:0060065 | uterus development | GO:0010718 | positive regulation of epithelial to mesenchymal transition |
| GO:0070374 | positive regulation of ERK1 and ERK2 cascade | GO:0042311 | vasodilation | GO:0030334 | regulation of cell migration |
| GO:0038063 | collagen-activated tyrosine kinase receptor signaling pathway | GO:0090170 | regulation of Golgi inheritance | GO:0071356 | cellular response to tumor necrosis factor |
| GO:0007275 | multicellular organism development | GO:0060978 | angiogenesis involved in coronary vascular morphogenesis | GO:0030224 | monocyte differentiation |
| GO:0006468 | protein phosphorylation | GO:0007613 | memory | GO:0071380 | cellular response to prostaglandin E stimulus |
| GO:0036323 | vascular endothelial growth factor receptor-1 signaling pathway | GO:0043401 | steroid hormone receptor signaling pathway | GO:0071363 | cellular response to growth factor stimulus |
| GO:0046777 | protein autophosphorylation | GO:0051091 | positive regulation of DNA-binding transcription factor activity | GO:0045471 | response to ethanol |
| GO:0035790 | platelet-derived growth factor receptor-alpha signaling pathway | GO:0051402 | neuron apoptotic process | GO:0030155 | regulation of cell adhesion |
| GO:0006338 | chromatin remodeling | GO:0071391 | cellular response to estrogen stimulus | GO:0008631 | intrinsic apoptotic signaling pathway in response to oxidative stress |
| GO:0038109 | Kit signaling pathway | GO:0010759 | positive regulation of macrophage chemotaxis | GO:0033280 | response to vitamin D |
| GO:0038145 | macrophage colony-stimulating factor signaling pathway | GO:0014911 | positive regulation of smooth muscle cell migration | GO:0042730 | fibrinolysis |
| GO:0031547 | brain-derived neurotrophic factor receptor signaling pathway | GO:0050673 | epithelial cell proliferation | GO:0030282 | bone mineralization |
| GO:0035791 | platelet-derived growth factor receptor-beta signaling pathway | GO:0031589 | cell-substrate adhesion | GO:0030593 | neutrophil chemotaxis |
| GO:0048012 | hepatocyte growth factor receptor signaling pathway | GO:0060324 | face development | GO:0045780 | positive regulation of bone resorption |
| GO:0009410 | response to xenobiotic stimulus | GO:0007204 | positive regulation of cytosolic calcium ion concentration | GO:0006955 | immune response |
| GO:0007165 | signal transduction | GO:0014823 | response to activity | GO:0030308 | negative regulation of cell growth |
| GO:0010629 | negative regulation of gene expression | GO:1900087 | positive regulation of G1/S transition of mitotic cell cycle | GO:0045785 | positive regulation of cell adhesion |
| GO:1902895 | positive regulation of miRNA transcription | GO:0048146 | positive regulation of fibroblast proliferation | GO:0009749 | response to glucose |
| GO:0007169 | cell surface receptor protein tyrosine kinase signaling pathway | GO:1905278 | positive regulation of epithelial tube formation | GO:0060252 | positive regulation of glial cell proliferation |
| GO:0042127 | regulation of cell population proliferation | GO:0043243 | positive regulation of protein-containing complex disassembly | GO:0048384 | retinoic acid receptor signaling pathway |
| GO:0001525 | angiogenesis | GO:0032310 | prostaglandin secretion | GO:0097696 | cell surface receptor signaling pathway via STAT |
| GO:0034614 | cellular response to reactive oxygen species | GO:0097009 | energy homeostasis | GO:0051092 | positive regulation of NF-kappaB transcription factor activity |
| GO:0045944 | positive regulation of transcription by RNA polymerase II | GO:2001243 | negative regulation of intrinsic apoptotic signaling pathway | GO:0009887 | animal organ morphogenesis |
| GO:0030574 | collagen catabolic process | GO:0097191 | extrinsic apoptotic signaling pathway | GO:0097194 | execution phase of apoptosis |
| GO:0043066 | negative regulation of apoptotic process | GO:0031623 | receptor internalization | GO:0031929 | TOR signaling |
| GO:0022617 | extracellular matrix disassembly | GO:2000378 | negative regulation of reactive oxygen species metabolic process | GO:0030520 | estrogen receptor signaling pathway |
| GO:0071222 | cellular response to lipopolysaccharide | GO:0120162 | positive regulation of cold-induced thermogenesis | GO:0030097 | hemopoiesis |
| GO:1904707 | positive regulation of vascular associated smooth muscle cell proliferation | GO:0043627 | response to estrogen | GO:0042752 | regulation of circadian rhythm |
| GO:0043491 | phosphatidylinositol 3-kinase/protein kinase B signal transduction | GO:0048015 | phosphatidylinositol-mediated signaling | GO:0007200 | phospholipase C-activating G protein-coupled receptor signaling pathway |
| GO:0071456 | cellular response to hypoxia | GO:0043303 | mast cell degranulation | GO:0090026 | positive regulation of monocyte chemotaxis |
| GO:0001666 | response to hypoxia | GO:0046697 | decidualization | GO:0002223 | stimulatory C-type lectin receptor signaling pathway |
| GO:0045429 | positive regulation of nitric oxide biosynthetic process | GO:0050829 | defense response to Gram-negative bacterium | GO:0050730 | regulation of peptidyl-tyrosine phosphorylation |
| GO:0032496 | response to lipopolysaccharide | GO:0032024 | positive regulation of insulin secretion | GO:0036092 | phosphatidylinositol-3-phosphate biosynthetic process |
| GO:0016310 | phosphorylation | GO:0010507 | negative regulation of autophagy | GO:0006809 | nitric oxide biosynthetic process |
| GO:0019221 | cytokine-mediated signaling pathway | GO:0007584 | response to nutrient | GO:0051493 | regulation of cytoskeleton organization |
| GO:0030307 | positive regulation of cell growth | GO:0034605 | cellular response to heat | GO:0048754 | branching morphogenesis of an epithelial tube |
| GO:0006508 | proteolysis | GO:0050853 | B cell receptor signaling pathway | GO:0001819 | positive regulation of cytokine production |
| GO:0032757 | positive regulation of interleukin-8 production | GO:0032094 | response to food | GO:0098609 | cell-cell adhesion |
| GO:0045766 | positive regulation of angiogenesis | GO:0007229 | integrin-mediated signaling pathway | GO:0040014 | regulation of multicellular organism growth |
| GO:0030198 | extracellular matrix organization | GO:1905564 | positive regulation of vascular endothelial cell proliferation | GO:0006509 | membrane protein ectodomain proteolysis |
| GO:0038083 | peptidyl-tyrosine autophosphorylation | GO:0050729 | positive regulation of inflammatory response | GO:0035331 | negative regulation of hippo signaling |
| GO:0006954 | inflammatory response | GO:0042552 | myelination | GO:0071466 | cellular response to xenobiotic stimulus |
| GO:0043065 | positive regulation of apoptotic process | GO:0010467 | gene expression | GO:0036120 | cellular response to platelet-derived growth factor stimulus |
| GO:0043536 | positive regulation of blood vessel endothelial cell migration | GO:0010573 | vascular endothelial growth factor production | GO:0009791 | post-embryonic development |
| GO:1902894 | negative regulation of miRNA transcription | GO:0051902 | negative regulation of mitochondrial depolarization | GO:1900227 | positive regulation of NLRP3 inflammasome complex assembly |
| GO:0045893 | positive regulation of DNA-templated transcription | GO:0060440 | trachea formation | GO:0002053 | positive regulation of mesenchymal cell proliferation |
| GO:0018105 | peptidyl-serine phosphorylation | GO:0043525 | positive regulation of neuron apoptotic process | GO:0032727 | positive regulation of interferon-alpha production |
| GO:0032760 | positive regulation of tumor necrosis factor production | GO:1901224 | positive regulation of non-canonical NF-kappaB signal transduction | GO:0035747 | natural killer cell chemotaxis |
| GO:0045937 | positive regulation of phosphate metabolic process | GO:0002931 | response to ischemia | GO:1990268 | response to gold nanoparticle |
| GO:0042593 | glucose homeostasis | GO:0045840 | positive regulation of mitotic nuclear division | GO:1902988 | neurofibrillary tangle assembly |
| GO:0000165 | MAPK cascade | GO:0010592 | positive regulation of lamellipodium assembly | GO:0002092 | positive regulation of receptor internalization |
| GO:0071276 | cellular response to cadmium ion | GO:0001774 | microglial cell activation | GO:1903076 | regulation of protein localization to plasma membrane |
| GO:0009612 | response to mechanical stimulus | GO:0071549 | cellular response to dexamethasone stimulus | GO:0002862 | negative regulation of inflammatory response to antigenic stimulus |
| GO:0031663 | lipopolysaccharide-mediated signaling pathway | GO:1903672 | positive regulation of sprouting angiogenesis | GO:0032740 | positive regulation of interleukin-17 production |
| GO:0046326 | positive regulation of D-glucose import | GO:0006974 | DNA damage response | GO:0010971 | positive regulation of G2/M transition of mitotic cell cycle |
| GO:0006979 | response to oxidative stress | GO:0042110 | T cell activation | GO:0008217 | regulation of blood pressure |
| GO:0032930 | positive regulation of superoxide anion generation | GO:0009611 | response to wounding | GO:0071346 | cellular response to type II interferon |
| GO:0000122 | negative regulation of transcription by RNA polymerase II | GO:0019722 | calcium-mediated signaling | GO:0001678 | intracellular glucose homeostasis |
| GO:0032731 | positive regulation of interleukin-1 beta production | GO:0045907 | positive regulation of vasoconstriction | GO:0002639 | positive regulation of immunoglobulin production |
| GO:0046677 | response to antibiotic | GO:0001935 | endothelial cell proliferation | GO:0000082 | G1/S transition of mitotic cell cycle |
| GO:0038096 | Fc-gamma receptor signaling pathway involved in phagocytosis | GO:0034612 | response to tumor necrosis factor | GO:0043123 | positive regulation of canonical NF-kappaB signal transduction |
| GO:0031295 | T cell costimulation | GO:0070301 | cellular response to hydrogen peroxide | GO:0030101 | natural killer cell activation |
| GO:0033138 | positive regulation of peptidyl-serine phosphorylation | GO:0007623 | circadian rhythm | GO:1990000 | amyloid fibril formation |
| GO:0032755 | positive regulation of interleukin-6 production | GO:0050679 | positive regulation of epithelial cell proliferation | GO:0022008 | neurogenesis |
| GO:0045821 | positive regulation of glycolytic process | GO:0038113 | interleukin-9-mediated signaling pathway | GO:0051602 | response to electrical stimulus |
| GO:0032722 | positive regulation of chemokine production | GO:0038110 | interleukin-2-mediated signaling pathway | GO:0048662 | negative regulation of smooth muscle cell proliferation |
| GO:0001938 | positive regulation of endothelial cell proliferation | GO:0030878 | thyroid gland development | GO:0006952 | defense response |
| GO:0046718 | symbiont entry into host cell | GO:0070372 | regulation of ERK1 and ERK2 cascade | GO:0032436 | positive regulation of proteasomal ubiquitin-dependent protein catabolic process |
| GO:0051239 | regulation of multicellular organismal process | GO:0030522 | intracellular receptor signaling pathway | GO:0070050 | neuron cellular homeostasis |
| GO:0006915 | apoptotic process | GO:0050852 | T cell receptor signaling pathway | GO:0007186 | G protein-coupled receptor signaling pathway |
| GO:0071260 | cellular response to mechanical stimulus | GO:0006909 | phagocytosis | GO:0038091 | positive regulation of cell proliferation by VEGF-activated platelet derived growth factor receptor signaling pathway |
| GO:0007254 | JNK cascade | GO:0045648 | positive regulation of erythrocyte differentiation | GO:0060374 | mast cell differentiation |
| GO:0070371 | ERK1 and ERK2 cascade | GO:0048008 | platelet-derived growth factor receptor signaling pathway | GO:0002366 | leukocyte activation involved in immune response |
| GO:0008542 | visual learning | GO:0035924 | cellular response to vascular endothelial growth factor stimulus | GO:0021697 | cerebellar cortex formation |
| GO:0031401 | positive regulation of protein modification process | GO:0045931 | positive regulation of mitotic cell cycle | GO:2000544 | regulation of endothelial cell chemotaxis to fibroblast growth factor |
| GO:2001028 | positive regulation of endothelial cell chemotaxis | GO:0032872 | regulation of stress-activated MAPK cascade | GO:0061308 | cardiac neural crest cell development involved in heart development |
| GO:0070102 | interleukin-6-mediated signaling pathway | GO:0008354 | germ cell migration | GO:0070269 | pyroptotic inflammatory response |
| GO:0016477 | cell migration | GO:0061042 | vascular wound healing | GO:0001958 | endochondral ossification |
| GO:0035556 | intracellular signal transduction | GO:0042473 | outer ear morphogenesis | GO:0048469 | cell maturation |
| GO:0014044 | Schwann cell development | GO:0048873 | homeostasis of number of cells within a tissue | GO:0060045 | positive regulation of cardiac muscle cell proliferation |
| GO:0030154 | cell differentiation | GO:0030225 | macrophage differentiation | GO:0035774 | positive regulation of insulin secretion involved in cellular response to glucose stimulus |
| GO:0035987 | endodermal cell differentiation | GO:0090050 | positive regulation of cell migration involved in sprouting angiogenesis | GO:0048705 | skeletal system morphogenesis |
| GO:0032869 | cellular response to insulin stimulus | GO:0031647 | regulation of protein stability | GO:0072593 | reactive oxygen species metabolic process |
| GO:0042531 | positive regulation of tyrosine phosphorylation of STAT protein | GO:0001501 | skeletal system development | GO:0097192 | extrinsic apoptotic signaling pathway in absence of ligand |
| GO:1904645 | response to amyloid-beta | GO:0042060 | wound healing | GO:0071345 | cellular response to cytokine stimulus |
| GO:2000573 | positive regulation of DNA biosynthetic process | GO:0045765 | regulation of angiogenesis | GO:0035633 | maintenance of blood-brain barrier |
| GO:0006006 | glucose metabolic process | GO:0042542 | response to hydrogen peroxide | GO:0010468 | regulation of gene expression |
| GO:0010595 | positive regulation of endothelial cell migration | GO:0051128 | regulation of cellular component organization | GO:0001662 | behavioral fear response |
| GO:0030168 | platelet activation | GO:0072540 | T-helper 17 cell lineage commitment | GO:0055074 | calcium ion homeostasis |
| GO:0120041 | positive regulation of macrophage proliferation | GO:2001240 | negative regulation of extrinsic apoptotic signaling pathway in absence of ligand | GO:1990090 | cellular response to nerve growth factor stimulus |
| GO:0038127 | ERBB signaling pathway | GO:0010634 | positive regulation of epithelial cell migration | GO:0071407 | cellular response to organic cyclic compound |
| GO:1900182 | positive regulation of protein localization to nucleus | GO:0002052 | positive regulation of neuroblast proliferation | GO:0060612 | adipose tissue development |
| GO:2000811 | negative regulation of anoikis | GO:0097190 | apoptotic signaling pathway | GO:0031669 | cellular response to nutrient levels |
| GO:0001541 | ovarian follicle development | GO:0071333 | cellular response to glucose stimulus | GO:0032008 | positive regulation of TOR signaling |
| GO:0008584 | male gonad development | GO:0001649 | osteoblast differentiation | GO:0001890 | placenta development |
| GO:0043524 | negative regulation of neuron apoptotic process | GO:2001237 | negative regulation of extrinsic apoptotic signaling pathway | GO:0043029 | T cell homeostasis |
| GO:0042307 | positive regulation of protein import into nucleus | GO:0008360 | regulation of cell shape | GO:0031100 | animal organ regeneration |
| GO:0002318 | myeloid progenitor cell differentiation | GO:0050830 | defense response to Gram-positive bacterium | GO:2001238 | positive regulation of extrinsic apoptotic signaling pathway |
| GO:0002042 | cell migration involved in sprouting angiogenesis | GO:0150078 | positive regulation of neuroinflammatory response | GO:0030182 | neuron differentiation |
| GO:0007507 | heart development | GO:0060397 | growth hormone receptor signaling pathway via JAK-STAT | GO:2000635 | negative regulation of primary miRNA processing |
| GO:0030163 | protein catabolic process | GO:0014912 | negative regulation of smooth muscle cell migration | GO:0032079 | positive regulation of endodeoxyribonuclease activity |
| GO:1904646 | cellular response to amyloid-beta | GO:0002446 | neutrophil mediated immunity | GO:0060571 | morphogenesis of an epithelial fold |
| GO:0071404 | cellular response to low-density lipoprotein particle stimulus | GO:2000117 | negative regulation of cysteine-type endopeptidase activity | GO:0070561 | vitamin D receptor signaling pathway |
| GO:2000641 | regulation of early endosome to late endosome transport | GO:0043542 | endothelial cell migration | GO:0060745 | mammary gland branching involved in pregnancy |
| GO:0048538 | thymus development | GO:0007595 | lactation | GO:0036005 | response to macrophage colony-stimulating factor |
| GO:0051403 | stress-activated MAPK cascade | GO:0016485 | protein processing | GO:0010646 | regulation of cell communication |
| GO:0030324 | lung development | GO:0032733 | positive regulation of interleukin-10 production | GO:0150003 | regulation of spontaneous synaptic transmission |
| GO:0051384 | response to glucocorticoid | GO:0008625 | extrinsic apoptotic signaling pathway via death domain receptors | GO:0042369 | vitamin D catabolic process |
| GO:0007166 | cell surface receptor signaling pathway | GO:0018107 | peptidyl-threonine phosphorylation | GO:0090258 | negative regulation of mitochondrial fission |
| GO:0006935 | chemotaxis | GO:0007405 | neuroblast proliferation | GO:0032227 | negative regulation of synaptic transmission, dopaminergic |
| GO:0031334 | positive regulation of protein-containing complex assembly | GO:2000648 | positive regulation of stem cell proliferation | GO:1905521 | regulation of macrophage migration |
| GO:0032355 | response to estradiol | GO:0010469 | regulation of signaling receptor activity | GO:0002551 | mast cell chemotaxis |
| GO:0030183 | B cell differentiation | GO:0001759 | organ induction | GO:0032765 | positive regulation of mast cell cytokine production |
| GO:0038133 | ERBB2-ERBB3 signaling pathway | GO:0030518 | nuclear receptor-mediated steroid hormone signaling pathway | GO:0033627 | cell adhesion mediated by integrin |
| GO:0051247 | positive regulation of protein metabolic process | GO:0043276 | anoikis | GO:0001937 | negative regulation of endothelial cell proliferation |
| GO:0008285 | negative regulation of cell population proliferation | GO:0060326 | cell chemotaxis | GO:0032212 | positive regulation of telomere maintenance via telomerase |
| GO:0010888 | negative regulation of lipid storage | GO:0050728 | negative regulation of inflammatory response | GO:0006695 | cholesterol biosynthetic process |
| GO:0150077 | regulation of neuroinflammatory response | GO:0071364 | cellular response to epidermal growth factor stimulus | GO:0033077 | T cell differentiation in thymus |
| GO:0098586 | cellular response to virus | GO:0007566 | embryo implantation | GO:0046330 | positive regulation of JNK cascade |
| GO:0090303 | positive regulation of wound healing | GO:0051918 | negative regulation of fibrinolysis | GO:0007409 | axonogenesis |
| GO:0090398 | cellular senescence | GO:0060333 | type II interferon-mediated signaling pathway | GO:0050850 | positive regulation of calcium-mediated signaling |
| GO:1903078 | positive regulation of protein localization to plasma membrane | GO:0033690 | positive regulation of osteoblast proliferation | GO:0009408 | response to heat |
| GO:0007259 | cell surface receptor signaling pathway via JAK-STAT | GO:0009755 | hormone-mediated signaling pathway | GO:0048144 | fibroblast proliferation |
| GO:0060020 | Bergmann glial cell differentiation | GO:0050796 | regulation of insulin secretion | GO:0050727 | regulation of inflammatory response |
| GO:0071492 | cellular response to UV-A | GO:0030316 | osteoclast differentiation | GO:0019233 | sensory perception of pain |
| GO:0048010 | vascular endothelial growth factor receptor signaling pathway | GO:0003007 | heart morphogenesis | GO:0043537 | negative regulation of blood vessel endothelial cell migration |
| GO:1900017 | positive regulation of cytokine production involved in inflammatory response | GO:0030890 | positive regulation of B cell proliferation | GO:0034599 | cellular response to oxidative stress |
| GO:0097421 | liver regeneration | GO:0031667 | response to nutrient levels | GO:0000902 | cell morphogenesis |
| GO:0010575 | positive regulation of vascular endothelial growth factor production | GO:0008283 | cell population proliferation | GO:0032728 | positive regulation of interferon-beta production |
| GO:0071230 | cellular response to amino acid stimulus | GO:0001878 | response to yeast | GO:0030501 | positive regulation of bone mineralization |
| GO:0045737 | positive regulation of cyclin-dependent protein serine/threonine kinase activity | GO:0048771 | tissue remodeling | GO:1901796 | regulation of signal transduction by p53 class mediator |
| GO:0060396 | growth hormone receptor signaling pathway | GO:0032966 | negative regulation of collagen biosynthetic process | GO:0051222 | positive regulation of protein transport |
| GO:0010763 | positive regulation of fibroblast migration | GO:0045087 | innate immune response | GO:0072584 | caveolin-mediated endocytosis |
| GO:0033628 | regulation of cell adhesion mediated by integrin | GO:0043409 | negative regulation of MAPK cascade | GO:0070945 | neutrophil-mediated killing of gram-negative bacterium |
| GO:0050927 | positive regulation of positive chemotaxis | GO:0042789 | mRNA transcription by RNA polymerase II | GO:0031622 | positive regulation of fever generation |
| GO:2000379 | positive regulation of reactive oxygen species metabolic process | GO:0010039 | response to iron ion | GO:0070849 | response to epidermal growth factor |
| GO:0007611 | learning or memory | GO:0002690 | positive regulation of leukocyte chemotaxis | GO:0045923 | positive regulation of fatty acid metabolic process |
| GO:0038166 | angiotensin-activated signaling pathway | GO:0030278 | regulation of ossification | GO:1901532 | regulation of hematopoietic progenitor cell differentiation |
| GO:0071498 | cellular response to fluid shear stress | GO:0010831 | positive regulation of myotube differentiation | GO:0061431 | cellular response to methionine |
| GO:2000352 | negative regulation of endothelial cell apoptotic process | GO:0048011 | neurotrophin TRK receptor signaling pathway | GO:0035264 | multicellular organism growth |
| GO:2001235 | positive regulation of apoptotic signaling pathway | GO:0048546 | digestive tract morphogenesis | GO:0071526 | semaphorin-plexin signaling pathway |
| GO:0071392 | cellular response to estradiol stimulus | GO:0016242 | negative regulation of macroautophagy | GO:0045892 | negative regulation of DNA-templated transcription |
| GO:0051000 | positive regulation of nitric-oxide synthase activity | GO:0002281 | macrophage activation involved in immune response | GO:0032735 | positive regulation of interleukin-12 production |
| GO:0002679 | respiratory burst involved in defense response | GO:1900034 | regulation of cellular response to heat | GO:0034097 | response to cytokine |
| GO:0031281 | positive regulation of cyclase activity | GO:0032682 | negative regulation of chemokine production | GO:0042177 | negative regulation of protein catabolic process |
| GO:0065008 | regulation of biological quality | GO:0034198 | cellular response to amino acid starvation | GO:0043434 | response to peptide hormone |
| GO:0070141 | response to UV-A | GO:0007596 | blood coagulation | GO:0048286 | lung alveolus development |
| GO:0035313 | wound healing, spreading of epidermal cells | GO:0050918 | positive chemotaxis | GO:0009411 | response to UV |
| GO:0051770 | positive regulation of nitric-oxide synthase biosynthetic process | GO:0042742 | defense response to bacterium | GO:0032526 | response to retinoic acid |
| GO:0030217 | T cell differentiation | GO:0007179 | transforming growth factor beta receptor signaling pathway |  |  |

**Tab. S4 71 cellular component (CC) of GO analysis**

| Term | Cellular component | Term | Cellular component | Term | Cellular component |
| --- | --- | --- | --- | --- | --- |
| GO:0043235 | receptor complex | GO:0005739 | mitochondrion | GO:0030424 | axon |
| GO:0005886 | plasma membrane | GO:0043231 | intracellular membrane-bounded organelle | GO:0031093 | platelet alpha granule lumen |
| GO:0005576 | extracellular region | GO:0031594 | neuromuscular junction | GO:0034682 | integrin alphav-beta1 complex |
| GO:0005737 | cytoplasm | GO:0005942 | phosphatidylinositol 3-kinase complex | GO:0097128 | cyclin D1-CDK4 complex |
| GO:0045121 | membrane raft | GO:0032587 | ruffle membrane | GO:0031234 | extrinsic component of cytoplasmic side of plasma membrane |
| GO:0005925 | focal adhesion | GO:0009925 | basal plasma membrane | GO:0005789 | endoplasmic reticulum membrane |
| GO:0031012 | extracellular matrix | GO:1904813 | ficolin-1-rich granule lumen | GO:0005768 | endosome |
| GO:0005615 | extracellular space | GO:0043209 | myelin sheath | GO:0005667 | transcription regulator complex |
| GO:0009986 | cell surface | GO:0044297 | cell body | GO:0005770 | late endosome |
| GO:0048471 | perinuclear region of cytoplasm | GO:0000785 | chromatin | GO:0005794 | Golgi apparatus |
| GO:0032991 | protein-containing complex | GO:0098794 | postsynapse | GO:0043197 | dendritic spine |
| GO:0005634 | nucleus | GO:0045202 | synapse | GO:1904090 | peptidase inhibitor complex |
| GO:0009897 | external side of plasma membrane | GO:0010008 | endosome membrane | GO:0005944 | phosphatidylinositol 3-kinase complex, class IB |
| GO:0005829 | cytosol | GO:0043025 | neuronal cell body | GO:0005899 | insulin receptor complex |
| GO:0016020 | membrane | GO:0005783 | endoplasmic reticulum | GO:0035867 | alphav-beta3 integrin-IGF-1-IGF1R complex |
| GO:0005901 | caveola | GO:0062023 | collagen-containing extracellular matrix | GO:0016323 | basolateral plasma membrane |
| GO:0090575 | RNA polymerase II transcription regulator complex | GO:0005769 | early endosome | GO:0042470 | melanosome |
| GO:0005943 | phosphatidylinositol 3-kinase complex, class IA | GO:0005764 | lysosome | GO:0005796 | Golgi lumen |
| GO:0035578 | azurophil granule lumen | GO:0016324 | apical plasma membrane | GO:0097180 | serine protease inhibitor complex |
| GO:0005788 | endoplasmic reticulum lumen | GO:0043005 | neuron projection | GO:0009898 | cytoplasmic side of plasma membrane |
| GO:0005911 | cell-cell junction | GO:0030027 | lamellipodium | GO:0097060 | synaptic membrane |
| GO:0005654 | nucleoplasm | GO:0035580 | specific granule lumen | GO:0031264 | death-inducing signaling complex |
| GO:0098978 | glutamatergic synapse | GO:0000791 | euchromatin | GO:0098793 | presynapse |
| GO:0070062 | extracellular exosome | GO:0070161 | anchoring junction |  |  |

**Tab. S5 150 molecular function (MF) of GO analysis**

| Term | Molecular function | Term | Molecular function | Term | Molecular function |
| --- | --- | --- | --- | --- | --- |
| GO:0004713 | protein tyrosine kinase activity | GO:0097110 | scaffold protein binding | GO:0051721 | protein phosphatase 2A binding |
| GO:0140801 | histone H2AXY142 kinase activity | GO:0005158 | insulin receptor binding | GO:0004712 | protein serine/threonine/tyrosine kinase activity |
| GO:0035401 | histone H3Y41 kinase activity | GO:0003707 | nuclear steroid receptor activity | GO:0001228 | DNA-binding transcription activator activity, RNA polymerase II-specific |
| GO:0019899 | enzyme binding | GO:0001784 | phosphotyrosine residue binding | GO:0070330 | aromatase activity |
| GO:0042802 | identical protein binding | GO:0008201 | heparin binding | GO:0001540 | amyloid-beta binding |
| GO:0004672 | protein kinase activity | GO:0001968 | fibronectin binding | GO:0005164 | tumor necrosis factor receptor binding |
| GO:0005009 | insulin receptor activity | GO:0034056 | estrogen response element binding | GO:0051425 | PTB domain binding |
| GO:0005018 | platelet-derived growth factor alpha-receptor activity | GO:0031994 | insulin-like growth factor I binding | GO:0070644 | vitamin D response element binding |
| GO:0005004 | GPI-linked ephrin receptor activity | GO:0008237 | metallopeptidase activity | GO:0160185 | phospholipase C activator activity |
| GO:0036332 | placental growth factor receptor activity | GO:0004705 | JUN kinase activity | GO:0004666 | prostaglandin-endoperoxide synthase activity |
| GO:0005020 | stem cell factor receptor activity | GO:0004707 | MAP kinase activity | GO:0016712 | oxidoreductase activity, acting on paired donors, with incorporation or reduction of molecular oxygen, reduced flavin or flavoprotein as one donor, and incorporation of one atom of oxygen |
| GO:0005011 | macrophage colony-stimulating factor receptor activity | GO:0004708 | MAP kinase kinase activity | GO:0004601 | peroxidase activity |
| GO:0060175 | brain-derived neurotrophic factor receptor activity | GO:0061629 | RNA polymerase II-specific DNA-binding transcription factor binding | GO:0072354 | histone H3T3 kinase activity |
| GO:0038062 | protein tyrosine kinase collagen receptor activity | GO:0043559 | insulin binding | GO:0044024 | histone H2AS1 kinase activity |
| GO:0008288 | boss receptor activity | GO:0030546 | signaling receptor activator activity | GO:0140855 | histone H3S57 kinase activity |
| GO:0005006 | epidermal growth factor receptor activity | GO:0030235 | nitric-oxide synthase regulator activity | GO:0035175 | histone H3S10 kinase activity |
| GO:0005019 | platelet-derived growth factor beta-receptor activity | GO:0003677 | DNA binding | GO:0044025 | histone H2BS14 kinase activity |
| GO:0005008 | hepatocyte growth factor receptor activity | GO:0003700 | DNA-binding transcription factor activity | GO:0072371 | histone H2AS121 kinase activity |
| GO:0005007 | fibroblast growth factor receptor activity | GO:0015026 | coreceptor activity | GO:1990244 | histone H2AT120 kinase activity |
| GO:0005010 | insulin-like growth factor receptor activity | GO:0003690 | double-stranded DNA binding | GO:0004694 | eukaryotic translation initiation factor 2alpha kinase activity |
| GO:0005021 | vascular endothelial growth factor receptor activity | GO:0046934 | 1-phosphatidylinositol-4,5-bisphosphate 3-kinase activity | GO:0072518 | Rho-dependent protein serine/threonine kinase activity |
| GO:0005005 | transmembrane-ephrin receptor activity | GO:0035005 | 1-phosphatidylinositol-4-phosphate 3-kinase activity | GO:0004711 | ribosomal protein S6 kinase activity |
| GO:0004714 | transmembrane receptor protein tyrosine kinase activity | GO:0000976 | transcription cis-regulatory region binding | GO:0044023 | histone H4S1 kinase activity |
| GO:0005524 | ATP binding | GO:0005518 | collagen binding | GO:0044022 | histone H3S28 kinase activity |
| GO:0019903 | protein phosphatase binding | GO:0071889 | 14-3-3 protein binding | GO:0004676 | 3-phosphoinositide-dependent protein kinase activity |
| GO:0004879 | nuclear receptor activity | GO:0016303 | 1-phosphatidylinositol-3-kinase activity | GO:0140823 | histone H2BS36 kinase activity |
| GO:0004175 | endopeptidase activity | GO:0019825 | oxygen binding | GO:0035979 | histone H2AXS139 kinase activity |
| GO:0005178 | integrin binding | GO:0001221 | transcription coregulator binding | GO:0004677 | DNA-dependent protein kinase activity |
| GO:0004252 | serine-type endopeptidase activity | GO:0001618 | virus receptor activity | GO:0140857 | histone H3T45 kinase activity |
| GO:0016301 | kinase activity | GO:0044877 | protein-containing complex binding | GO:0035403 | histone H3T6 kinase activity |
| GO:0008233 | peptidase activity | GO:0051117 | ATPase binding | GO:0035402 | histone H3T11 kinase activity |
| GO:0001223 | transcription coactivator binding | GO:0050661 | NADP binding | GO:0004679 | AMP-activated protein kinase activity |
| GO:0004222 | metalloendopeptidase activity | GO:0005516 | calmodulin binding | GO:0050692 | DNA binding domain binding |
| GO:0019901 | protein kinase binding | GO:0046872 | metal ion binding | GO:0090722 | receptor-receptor interaction |
| GO:0005515 | protein binding | GO:0051879 | Hsp90 protein binding | GO:0030284 | nuclear estrogen receptor activity |
| GO:0004674 | protein serine/threonine kinase activity | GO:0019955 | cytokine binding | GO:0030331 | nuclear estrogen receptor binding |
| GO:0043560 | insulin receptor substrate binding | GO:0046982 | protein heterodimerization activity | GO:0001530 | lipopolysaccharide binding |
| GO:0008270 | zinc ion binding | GO:0008395 | steroid hydroxylase activity | GO:0042056 | chemoattractant activity |
| GO:0005102 | signaling receptor binding | GO:0008083 | growth factor activity | GO:0030295 | protein kinase activator activity |
| GO:0005496 | steroid binding | GO:0051087 | protein-folding chaperone binding | GO:0038085 | vascular endothelial growth factor binding |
| GO:0002020 | protease binding | GO:0140297 | DNA-binding transcription factor binding | GO:0005497 | androgen binding |
| GO:0020037 | heme binding | GO:1990782 | protein tyrosine kinase binding | GO:0019960 | C-X3-C chemokine binding |
| GO:0106310 | protein serine kinase activity | GO:0003682 | chromatin binding | GO:0043125 | ErbB-3 class receptor binding |
| GO:0042169 | SH2 domain binding | GO:0043274 | phospholipase binding | GO:0048018 | receptor ligand activity |
| GO:0042803 | protein homodimerization activity | GO:0044389 | ubiquitin-like protein ligase binding | GO:0005125 | cytokine activity |
| GO:0004715 | non-membrane spanning protein tyrosine kinase activity | GO:0000978 | RNA polymerase II cis-regulatory region sequence-specific DNA binding | GO:0141038 | phosphatidylinositol 3-kinase activator activity |
| GO:0031625 | ubiquitin protein ligase binding | GO:0031711 | bradykinin receptor binding | GO:0031730 | CCR5 chemokine receptor binding |
| GO:0043565 | sequence-specific DNA binding | GO:0062181 | 1-alpha,25-dihydroxyvitamin D3 23-hydroxylase activity | GO:0005138 | interleukin-6 receptor binding |
| GO:0043548 | phosphatidylinositol 3-kinase binding | GO:0140677 | molecular function activator activity | GO:0050693 | LBD domain binding |
| GO:0019838 | growth factor binding | GO:0046875 | ephrin receptor binding | GO:0019902 | phosphatase binding |
